# A comprehensive transcription factor and DNA-binding motif resource for the construction of gene regulatory networks in *Botrytis cinerea* and *Trichoderma atroviride*

**DOI:** 10.1016/j.csbj.2021.11.012

**Published:** 2021-11-18

**Authors:** Consuelo Olivares-Yañez, Evelyn Sánchez, Gabriel Pérez-Lara, Aldo Seguel, Pamela Y. Camejo, Luis F. Larrondo, Elena A. Vidal, Paulo Canessa

**Affiliations:** a ANID – Millennium Science Initiative Program – Millennium Institute for Integrative Biology (iBio), Avda. Libertador Bernardo O’Higgins 340, Santiago, Chile; b Centro de Biotecnologia Vegetal, Universidad Andres Bello, Republica 330, Santiago, Chile; c Centro de Genomica y Bioinformatica, Facultad de Ciencias, Universidad Mayor, Camino la Pirámide 5750, Huechuraba, Santiago, Chile; d Escuela de Biotecnologia, Facultad de Ciencias, Universidad Mayor, Camino la Pirámide 5750, Huechuraba, Santiago, Chile; e Departamento de Genetica Molecular y Microbiologia, Facultad de Ciencias Biologicas, Pontificia Universidad Catolica de Chile, Avda. Libertador Bernardo O’Higgins 340, Santiago, Chile

**Keywords:** Botrytis cinerea, Trichoderma atroviride, Transcription factors, Gene Regulatory Network, Mycoparasitism

## Abstract

*Botrytis cinerea* and *Trichoderma atroviride* are two relevant fungi in agricultural systems. To gain insights into these organisms’ transcriptional gene regulatory networks (GRNs), we generated a manually curated transcription factor (TF) dataset for each of them, followed by a GRN inference utilizing available sequence motifs describing DNA-binding specificity and global gene expression data. As a proof of concept of the usefulness of this resource to pinpoint key transcriptional regulators, we employed publicly available transcriptomics data and a newly generated dual RNA-seq dataset to build context-specific *Botrytis* and *Trichoderma* GRNs under two different biological paradigms: exposure to continuous light and *Botrytis-Trichoderma* confrontation assays. Network analysis of fungal responses to constant light revealed striking differences in the transcriptional landscape of both fungi. On the other hand, we found that the confrontation of both microorganisms elicited a distinct set of differentially expressed genes with changes in *T. atroviride* exceeding those in *B. cinerea*. Using our regulatory network data, we were able to determine, in both fungi, central TFs involved in this interaction response, including TFs controlling a large set of extracellular peptidases in the biocontrol agent *T. atroviride*. In summary, our work provides a comprehensive catalog of transcription factors and regulatory interactions for both organisms. This catalog can now serve as a basis for generating novel hypotheses on transcriptional regulatory circuits in different experimental contexts.

## Graphical abstract

**Figure:**
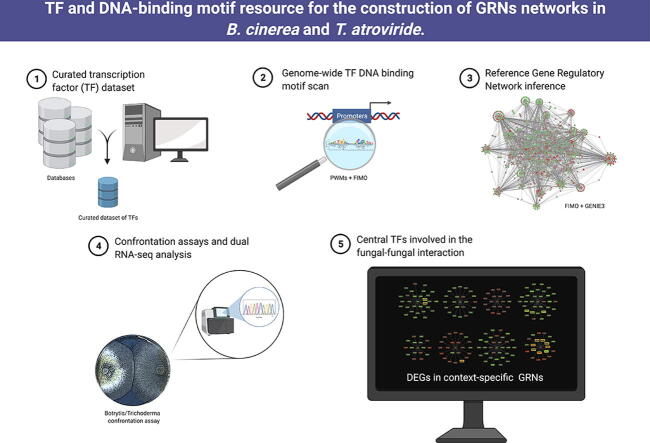

## 1 Introduction

Living organisms constantly need to integrate external biotic and abiotic cues to adapt and survive in a changing environment. Furthermore, external signals must be integrated with internal developmental programs to generate a response. Part of this response is driven by changes at the gene expression level, where transcription factors (TFs) - among other regulatory molecules - play a pivotal role. TFs are proteins characterized by the presence of one or more DNA-binding domains (DBDs) that recognize specific motifs in DNA sequences. Different experimental high-throughput approaches are used to determine the DNA binding preferences of these molecules, such as Protein Binding Microarrays (PBMs), SELEX-Seq, DAP-Seq [1], [2], [3], or ChIP-Seq. Importantly, DNA binding motifs for TFs with no experimental data can be inferred based on sequence similarity. For instance, Weirauch et al. [4] used PBMs to directly determine motifs for more than 1000 TFs over 130 species and used this data to infer motifs for 58,000 additional TFs. Information of TFs, as well as their DNA binding motifs, are collected in different databases, such as Transfac [5], JASPAR [6], HOCOMOCO [7], or CisBP [4]. In these, the DNA binding specificity of a given TF is represented as a Position Weight Matrix (PWM) that summarizes the observed frequencies within the motif of each nucleotide at each position. Therefore, these PWMs can be used to scan genomic sequences to identify genome-wide TF binding sites (TFBSs), and thus, putative target genes for the corresponding TF. This TF-target relationship is crucial for understanding transcriptional control mechanisms underlying most biological processes.

Genome-scale characterization of regulatory interactions is one of the goals of systems biology. One approach to describe the complex interactions between TFs and their target genes is by building Gene Regulatory Network (GRN) models, which are graphical representations denoting TFs and non-TF genes as nodes connected with edges depicting the regulatory interconnections [8]. Although TFBS prediction using PWMs is a valuable resource, especially for organisms with few experimental data, more sophisticated GRN inference approaches benefit from high-throughput gene expression data to derive possible regulatory interactions [9]. These approaches include statistical dependencies between gene expression patterns (as correlations or mutual information), boolean logic functions, and Bayesian or regression models, among others. Integration of TFBS knowledge and GRN modeling algorithms have allowed the reconstruction of GRNs in several species of bacteria [10], [11], plants [12], [13], [14], animals [15], [16], [17], [18], and fungi [19], [20], [21], [22], [23], [24].

In fungal systems, the reconstruction of GRNs has been limited by the availability of experimentally characterized TF-target interactions and high-throughput gene expression datasets. Thus, most GRNs come from studies in the model species *Saccharomyces cerevisiae*, *Aspergillus nidulans*, and *Neurospora crassa* [25], [26], [27], [28], [29], [30]. To overcome these limitations, recent studies have adopted homology-based approaches, in which TF-target relationships are inferred from experimentally validated interactions occurring between orthologous TFs, to dissect GRNs in lesser-known species such as *Ustilago maydis* and *Penicillium* spp. [23], [24]. Information obtained from these fungal GRNs has allowed the identification of TFs and gene modules controlling essential fungal processes including, but not limited to, sexual reproduction, degradation of complex carbon compounds, production of mycotoxins, cell death, and stress responses.

Fungal organisms constitute one of the largest groups of plant pathogens, and the emergence of new fungicide-resistant strains is compromising human food security and wildlife biodiversity [31]. On the other hand, fungi can also benefit plant growth and productivity, improve nutrient uptake, generate plant growth regulators, boost the plant immune response, or act as biocontrollers of harmful pathogenic fungal organisms. Some fungal species, including mycorrhizae or other rhizospheric or endophytic fungi, are used as biofertilizers to promote crop productivity. However, despite the evident relevance of having reference GRNs to study organismal function and responses, to date, no genome-wide GRNs are available for fungal species with either negative or positive impact on agriculture and crop production, with only small-scale networks reported for the plant pathogen *Fusarium graminareum* [32], [33]. Among detrimental fungal phytopathogens, the grey mold fungus *Botrytis cinerea* occupies a position of distinction, being ranked as the second most important fungal phytopathogen worldwide [34] while among fungal biocontroller agents, *Trichoderma* has been recently syndicated as the fungal genus with the greatest biocontrol potential [35].

*B. cinerea* can infect over 1000 plant species, including numerous crops. It has a predominant necrotrophic lifestyle, co‐opting the host programmed cell death response to achieve infection [36]. *B. cinerea* is troublesome to control in agricultural fields due to its diverse attack modes and a broad range of hosts. Besides, it can survive as mycelia, conidia, or sclerotia under extended unfavorable periods, and the appearance of fungicide‐resistant isolates has been well documented [34], [37], [38]. Recent investigations have provided evidence of dynamic events occurring during the progression of the infection, involving protein secretion of cell-wall degrading (CWD) enzymes, bidirectional microvesicle sRNAs exchange with plants, and time-of-the-day dependent events that impact virulence [39], [40], [41], [42], [43].

Biological control agents like *Trichoderma spp*., a natural antagonist of *B. cinerea* and other fungal phytopathogens, could reduce or prevent the use of environmentally unfriendly chemical pesticides, avoiding the upsurge of new fungicide-resistant strains. *T. atroviride* is a fast-growing ascomycete that can be found in soil as free-living or associated with plants, favoring advantageous outcomes such as plant growth, strengthening abiotic stress tolerance, and enhancing resistance to pathogens [44], [45]. Through mycoparasitism, antibiosis, and competition, *T. atroviride* obtains nutrients employing CWD enzymes, among diverse strategies that include antimicrobial compounds [38]. Its success as a biocontroller can also be attributed to its ability to survive in unfavorable conditions, its high reproductive capacity, efficient nutrient utilization, and a strong mycoparasitic response [44], [46], [47], [48], [49].

To leverage the molecular understanding of the events shaping transcriptional responses in both *B. cinerea* and *T. atroviride*, we constructed reference GRNs employing their latest genome assemblies. We inferred whole-genome TFBS by first compiling a manually curated TF dataset for both fungi. These networks were refined using public high-throughput gene expression data. To assess the relevance and applicability of these GRNs, and considering the major transcriptional impact of light on fungal physiology [50], we integrated gene expression datasets from *B. cinerea* and *T. atroviride* grown in constant light and darkness with our reference GRN to build light-dependent GRNs for both organisms. In addition, we carried out fungal confrontation experiments between the biocontroller agent and the phytopathogen to build confrontation-dependent GRNs. These results provide both fungal communities with an unprecedented resource, facilitating a standardized strategy to formulate data-derived hypotheses.

## 2 Materials and Methods

### 2.1 Identification of transcription factors in *B. cinerea* and *T. atroviride* from proteome data

Protein sequences for *B. cinerea* and *T. atroviride* (genome assemblies ASM83294v1 and TRIAT_v2.0, respectively) were retrieved from EnsemblFungi [51]. The sequences were queried using InterProScan (V5.44-79) [52] to determine associated InterPro and PFAM IDs. To determine proteins that correspond to TFs, a list of IPR and PFAM IDs that have been previously utilized to determine proteins that correspond to TFs was gathered from information obtained from AnimalTFDB, PlantTFDB [53], [54] as well as from a previous report of fungal TF DBDs [55] (Supplementary File 1). Proteins from *B. cinerea* and *T. atroviride* containing at least one of these IDs were selected for further analysis. In parallel, a custom Hidden Markov Model (HMM) profile for TF DBDs was generated (Supplementary File 2) and used to scan the proteomes using hmmsearch (HMMER V.3.3.1) [56]. Proteins selected as candidate TFs according to the InterProScan and/or hmmsearch analysis were functionally annotated using information from BLAST2GO [57] and FungiFun [58]. Finally, manual curation of the sequences was performed. This procedure consisted of a careful case-by-case revision of each protein assigned as TF, based on its BLAST2GO name and description and FungiFun information (name and GO annotation). Proteins having annotations related to enzymatic activities (e.g., dehydrogenases, kinases, acetyltransferases), proteins related to molecular processes other than transcription (e.g., DNA replication, DNA repair, splicing, translation), proteins involved in transcription control other than TFs (e.g., basal transcription factors, RNA polyadenylation factors), subunits of chromatin remodeling complexes, actin-binding proteins, RNA binding proteins, as well as centromere, histone-related, ribosomal, scaffold, transporter, and tRNA related proteins were discarded as potential TFs. All the annotations and criteria employed in each case are indicated in Supplementary File 3 (for *B. cinerea* and *T. atroviride,* respectively).

### 2.2 Position Weight Matrix (PWM) assignment describing the DNA binding preference of each TF

To assign a DNA binding motif to each identified and manually-curated TF, the “Protein Scan” web tool at CisBP (http://cisbp.ccbr.utoronto.ca/TFTools.php) was used to query the full-length protein sequences. The highest scoring motif considered for each TF was selected. Only motifs belonging to fungal TFs were retrieved. Therefore, motifs derived from TFs of non-fungal organisms were discarded. In the case of TFs with more than one DBD, we retrieved PWMs describing both DBD preferences by searching each DBD at the “Protein Scan” web tool (Supplementary File 4).

For TF sequences with no identified motif in the CisBP “Protein Scan” web tool [4], an orthogroup classification was conducted. For this, a custom set of proteins consisting only of TFs was generated, including those from *S. cerevisiae*, *A. nidulans,* and *N. crassa* (S288C Sacce1, AspGD Aspnid1, and OR74A v2.0 Neucr2 proteomes, respectively)*,* species that harbor the largest dataset of fungal TFs with direct experimental determination of DNA binding preferences [4]*. B. cinerea* and *T. atroviride* TFs were classified into different TF orthogroups by first performing an all-against-all BLASTp analysis followed by automatic orthogroup definition carried out by OrthoFinder software (v 2.4.0) [59]. Based on this classification, PWMs were assigned to this particular group of TFs.

Finally, PWMs corresponding to each assigned motif were obtained from CisBP. Each TF-PWM pair (when available) for both *B. cinerea* and *T. atroviride* is described in Supplementary Files 4 and 5.

### 2.3 Genome-wide mapping of TF binding sites in promoter regions

To determine putative target genes for the *B. cinerea* and *T. atroviride* TFs, promoter sequences for both fungi were obtained by extracting a genomic sequence of 1000 bp upstream from the transcription start site (TSS) of each gene. TSS information was obtained from gene annotation files from both fungi, available on EnsemblFungi (ASM83294v1 and TRIAT_v2.0 for *B. cinerea* and *T. atroviride,* respectively). PWMs for each TF were then used to scan promoter sequences using the Find Individual Motif Occurrences (FIMO) tool from the MEME Suite (v 4.11.2) [60], employing default parameters (p-value < 1 × 10e^-4^).

### 2.4 Fungal strains and culture conditions employed in confrontation assays

Strain B05.10 of *B. cinerea* Pers. Fr. [*Botryotinia fuckeliana* (de Bary) Whetzel] was originally isolated from *Vitis vinifera* (Germany) [61], whereas strain IMI206040 of *T. atroviride* was first isolated from a plum tree in southern Sweden [62]. Both fungal strains were maintained in Petri plates containing potato dextrose agar (PDA, Becton Dickinson). Confrontation assays were performed as described [63]. Briefly, a 6 mm diameter mycelial plug of each fungus was placed on opposite sides of a PDA-containing Petri dish, allowing fungi to grow. Control plates were inoculated only with *B. cinerea* or *T. atroviride.* To facilitate mycelia harvesting, the culture media was covered with a cellophane overlay. Cultures were incubated for 3 days in Percival incubators (Percival Scientific, U.S.A.) at 20 °C in constant light (light intensity up to 100 Âµmol/m^2^/s; wavelength 400–720 nm) until both fungi reached each other (Supplementary Fig. 1). Constant light was used since circadian regulation and light have a significant impact on *B. cinerea* and *T. atroviride* physiology and interaction ([64], [42], and unpublished results).

### 2.5 High-quality RNA extraction, preparation of Illumina libraries, and sequencing

Approximately 10 mg of tissue was collected from the *Botrytis-Trichoderma* interaction zone (Supplementary Fig. 1) or the *B. cinerea* and *T. atroviride* plates, dried, and snap-frozen in liquid nitrogen. Frozen mycelia were ground to powder, and total RNA was extracted using TRIzol reagent (cat n° 15596026, Invitrogen) [65] according to the manufacturer’s instructions. Briefly, 1 ml of TRIzol reagent was added to each sample and processed as reported earlier [66]. Total RNA quantity and quality were determined using a NanoDrop spectrophotometer (Thermo Scientific) followed by fluorescence-based capillary electrophoresis (Fragment Analyzer; Advanced Analytical). The RNA Integrity Number (RIN) of all analyzed samples was higher than 7. Thereafter, poly-A-containing mRNA was obtained from the aforementioned total RNA. According to the manufacturer’s instructions, Illumina libraries were constructed using the TruSeq Stranded RNA Sample Preparation Kit (cat n° 20020595, Illumina). Library integrity and size were assessed by fluorescence-based capillary electrophoresis. Sequencing of the libraries was carried out in a HiSeq2000 sequencer, using 150 bp Paired-End mode (Macrogen Inc., Seoul, South Korea). Three independent biological replicates were sequenced for each condition.

### 2.6 Analysis of differential gene expression

Low-quality reads and adapter sequences were filtered out from FASTQ files using BBDuk (https://sourceforge.net/projects/bbmap/) (v38.18; ktrim = r k = 23 mink = 11 hdist = 2 qtrim = rl trimq = 10 ftm = 5 maq = 15 minlength = 50 tpe tbo). Filtered reads were mapped to each fungal genome using HiSat2 [67], with default parameters. Mapped read counts for each gene were determined employing Rsubread [68]. For determining differentially expressed genes (DEGs) between control and treatment conditions, likelihood ratio tests (LRTs) were performed using DESeq2 [69]. A Benjamini-Hochberg method for multiple testing was applied to adjust p‐values [70], and genes were filtered based on adjusted p‐values < 0.05 and log2 (fold-change) greater than 1.

### 2.7 Gene regulatory network construction

To generate a reference GRN for *B. cinerea* and *T. atroviride*, we combined the TF-target information obtained by FIMO with regulatory interactions predicted by GENIE3 (v 1.14.0, running in R 4.1.0), a widely used expression-based GRN inference algorithm [9]. To build the GENIE3 GRNs, we first generated a curated RNA-Seq dataset for *B. cinerea* and *T. atroviride* from data obtained from the NCBI Sequence Read Archive (SRA) (Supplementary File 8). For this purpose, the entire dataset was quality-filtered using BBDuk, as mentioned above. Only samples with at least 1 million reads mapped to each fungal genome were considered for downstream analysis. Gene expression in the datasets was determined by pseudoalignment of the reads to *B. cinerea* or *T. atroviride* transcripts using Kallisto (v.0.44.0) [71] under standard settings for Single End (–single -b 100 -l 100 -s 20) or Paired-End (-b 100) reads. The gene expression matrices, including the newly generated transcriptomic data obtained from confrontation assays reported herein, were used as input for GENIE3, and regulatory interactions were generated using default settings. The reference networks contain FIMO TF-target interactions corresponding to the 20% highest scoring TF-target pairs reported by GENIE3. A TF-target interaction was considered if at least one TF binding site (TFBS) was found in the promoter region of each gene. The resulting networks were visualized using Cytoscape (v 3.8.0) [72], and their properties were determined using the NetworkAnalyzer tool. Constructed clusters of genes in the reference network and sub-networks were determined using ClusterMaker2 [73], employing Community Clustering (GLay).

To test our reference GRNs, we obtained the list of DEGs for six loss-of-function TF mutants for *B. cinerea* and *T. atroviride*. In the case of previously published microarray experiments, we retrieved DEGs between each mutant and the wild-type genotype from each publication [74], [75], [76], [77]. In the case of RNA-seq experiments, data was downloaded [78] and analyzed as described in the former section. After that, we compared the lists of predicted direct targets for each TFs to the list of DEGs in the mutant genotype. Significant overlap between lists was calculated with the R package “GeneOverlap” (version 1.30.0; p < 0.05, Fisher’s exact test) (http://shenlab-sinai.github.io/shenlab-sinai/).

### 2.8 Functional annotation of genes and functional term enrichment analysis

To perform functional term enrichment analyses of the data, we first carried out a whole-genome functional annotation of *B. cinerea* and *T. atroviride* using the BLAST2GO functional annotation pipeline [57] to complement the information available in FungiFun [58]. Briefly, the whole protein dataset of both fungi was retrieved from EnsemblFungi to conduct a BLASTp search against the NCBI non-redundant (NR) database (fungi subset) employing BLAST2GO default parameters (OmicsBox software v1.4.11). An InterPro domain and an eggNOG database search were finally combined in BLAST2GO as described [57]. For each fungus, a gene annotation file (gaf) was constructed and used in BiNGO (v.3.0.3) [79] to determine Gene Ontology (GO) enriched terms. Alternatively, we also used the FungiFun web tool software [58]. A false discovery rate (FDR) correction (p < 0.05) was applied to the overrepresented GO terms after performing a hypergeometric test in both tools.

### 2.9 RNAseq datasets analyses

Publicly available RNA-Seq data was downloaded from the NCBI SRA database. In the case of the publicly available experiments in constant light for *T. atroviride*, this corresponds to the SRA accession number SRP069026 [80], while in the case of *B. cinerea* to accession number SRP235144. The new data reported herein was deposited in SRA with the following accession number: PRJNA756518.

## 3 Results and discussion

### 3.1 Defining the repertoire of transcription factors for *B. cinerea* and *T. atroviride* and their respective DNA-binding preferences

**Fig. 1:**
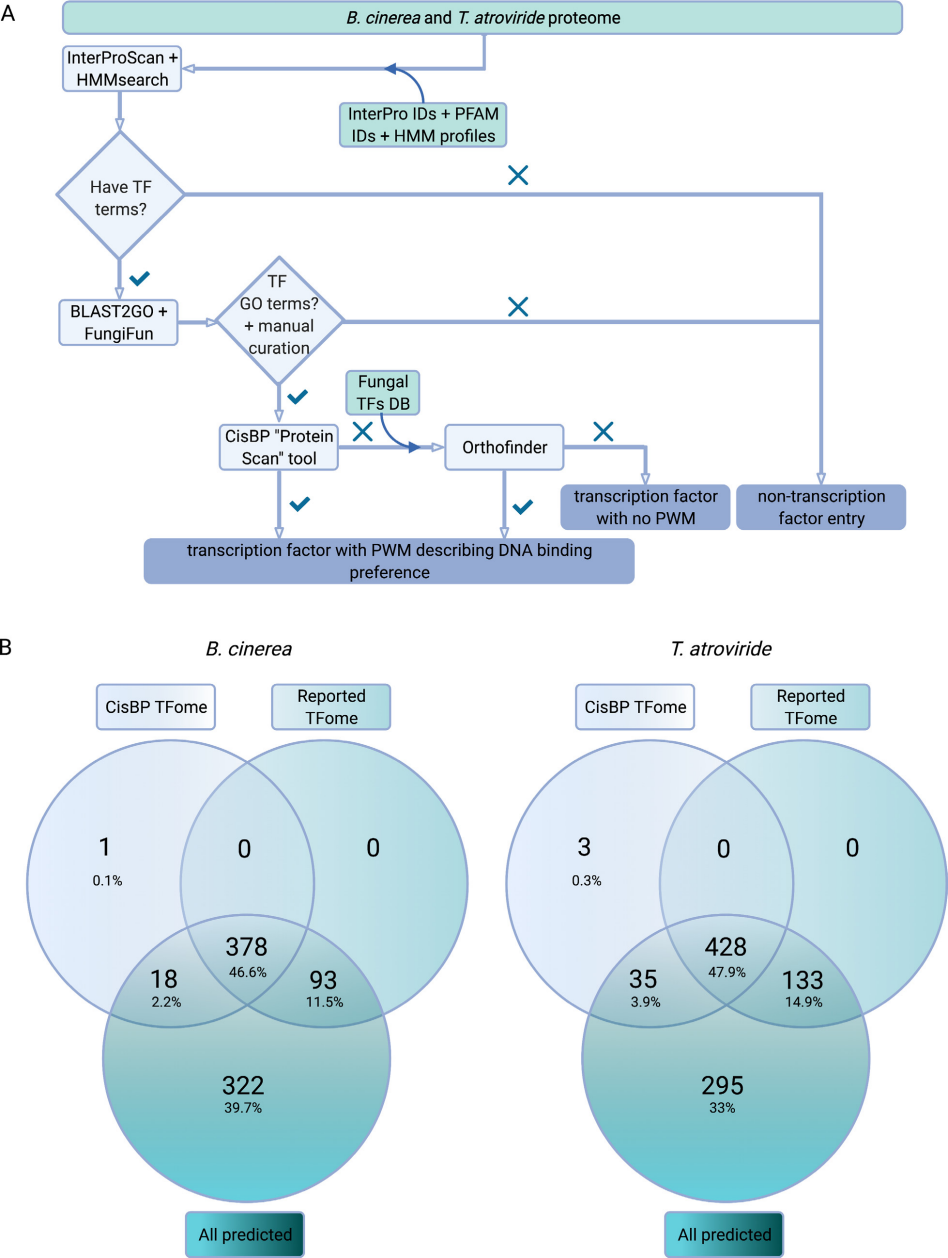
Transcription factor annotation, DNA binding motif assignment, and manual curation process overview for *B. cinerea* and *T. atroviride*. (A) Schematic representation of the bioinformatics pipeline used to generate a dataset of TFs with their assigned PWMs describing their putative DNA-binding motifs for both fungi. Green boxes denote databases (DBs), while light blue boxes indicate employed software. Curved arrows show DBs feed into software. Romboids represent critical decision steps. The final outputs (blue boxes) are displayed at the bottom. (B) Venn diagrams of the data presented in (A) showing the overlap of TFs before manual curation (“All predicted”), the “CisBP TFome”, and the final dataset of curated TFs (“Reported TFome”) described herein. (For interpretation of the references to colour in this figure legend, the reader is referred to the web version of this article.)

Despite the significant increase in the number of fully sequenced fungal genomes surpassing 1000 species several years ago [81] and the major regulatory significance of TFs across phyla, there is no current fungal initiative addressing the systematic categorization of these regulatory proteins. For example, the last update of the Fungal Transcription Factor Database [82] was more than 10 years ago, in July 2009. Thus, to start building reference GRNs for *B. cinerea* and *T. atroviride*, we first established a manually-curated full repertoire of TFs (TFome) for both fungi by *de novo* annotation of their reported proteins using different protein annotation tools and criteria, as depicted in Fig. 1A and as explained in the Materials and Methods section. To conduct the annotation of TFs, we generated a list of InterPro and PFAM identifiers, as well as HMM profiles corresponding to DBDs (Supplementary Files 1 and 2), not considering IDs associated with proteins related to the basal transcription machinery or RNA binding that have been previously included in other studies [23], [55]. Classification of fungal protein domains predicted 811 and 891 TFs for *B. cinerea* and *T. atroviride*, respectively (Supplementary File 3). These numbers significantly differ from previous TF annotations, including the CisBP database (397 and 466 for *B. cinerea* and *T. atroviride*, respectively) and the work of Shelest, 2017, which reports 411 and 577 TFs, for *B. cinerea* and *T. atroviride*, respectively. Thus, a manual inspection of the predicted TF list was undertaken to filter proteins erroneously assigned as TFs, as described in Materials and Methods (e.g., components of basal transcription factors such as subunits of TFIID, RNA polyadenylation, splicing factors, among others, Supplementary File 3). Manual inspection of each sequence generated a final curated TF list consisting of 471 and 561 sequences for *B. cinerea* and *T. atroviride*, respectively (Supplementary File 5). For *B. cinerea,* 378 of these TFs were also reported in CisBP, while 93 additional TFs were found by our annotation pipeline (Fig. 1B). In addition, 18 CisBP-annotated protein sequences were manually discarded. For example, Bcin10g03410, annotated as TF in CisBP, has a CENP-B N-terminal DNA-binding domain. However, its function is related to centromere organization [83], [84]. For *T. atroviride,* 428 TFs are also found in CisBP, and 133 new TFs were identified by our procedure. After manual curation, 35 sequences previously defined as TFs in CisBP were discarded (Fig. 1B). On the aggregate, at least for *B. cinerea*, the number of TFs found is similar to other reported Ascomycota TFomes (366 TFs on average [85]).

**Fig. 2:**
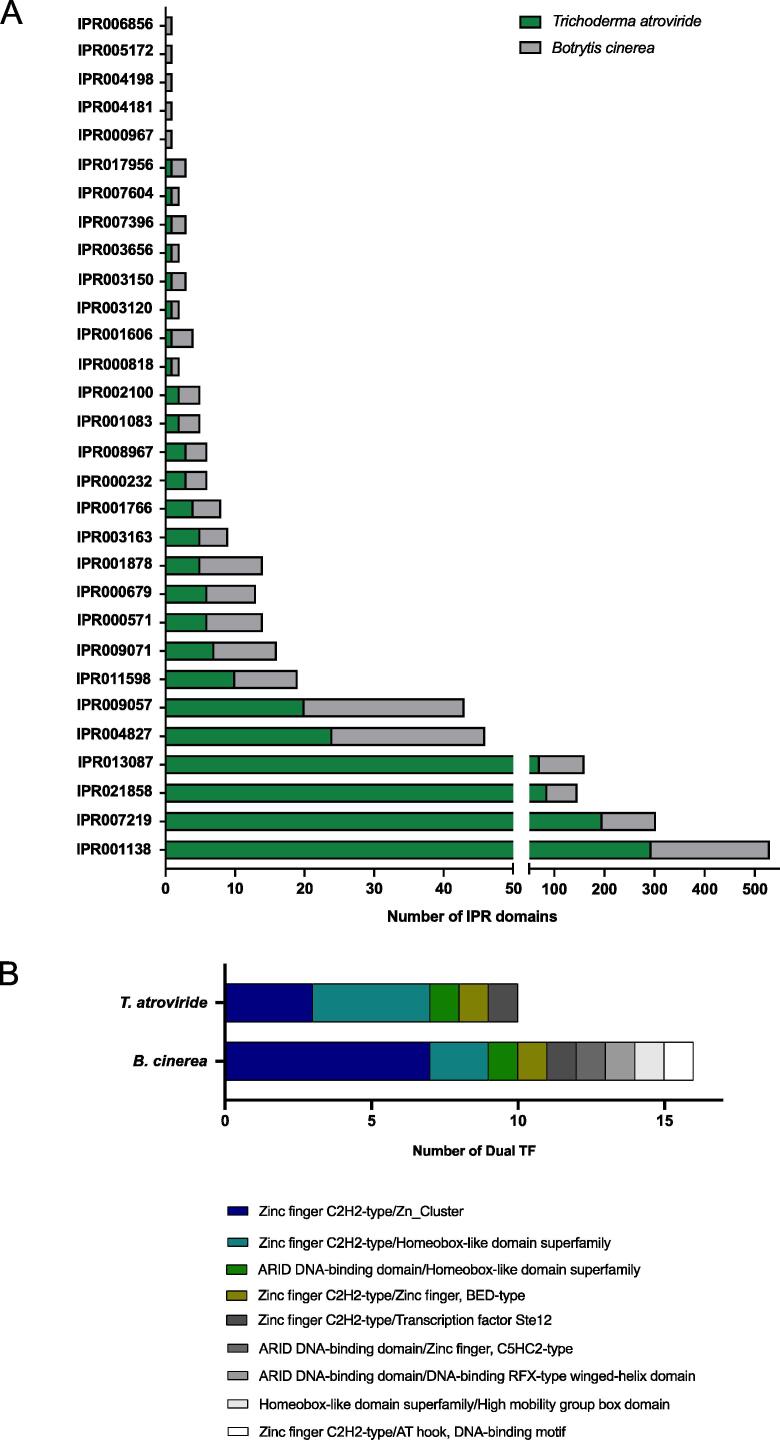
Distribution of InterPro domains observed among *B. cinerea* and *T. atroviride* curated transcription factors. (A) Based on an InterProScan search, 30 IPR identifiers were recognized in the case of *B. cinerea* while 25 in *T. atroviride.* (B) Combination of the DBDs of dual-specificity TFs identified in *B. cinerea* and *T. atroviride.* Each DBD combination is described in Table 2.

Based on InterPro IDs, we found a total of 30 and 25 TF families for *B. cinerea* and *T. atroviride*, respectively (Table 1). Protein family classification based on PFAM IDs is provided in Supplementary Table 1. The most represented TF domains in both fungi correspond to the “Zn(2)-C6 fungal-type” DBD (IPR001138), also known as the Zn cluster domain (38.2% and 39.1% of all the TF domains in *B. cinerea* and *T. atroviride*, respectively) (Table 1; Fig. 2A). Consistent with this result, the expansion of the Zn cluster family has been previously reported as a feature of Ascomycete TFomes [55], [85]. Zn cluster family members are found in all fungal species [86], [87], [88]. Due to their prevalence and high representation in fungal organisms, Zn cluster TFs participate in crucial fungal pathways, including sugar and nitrogen metabolism, respiration, vitamin synthesis, mitosis and meiosis, chromatin remodeling, stress response, and multidrug resistance, among others [89]. For instance, in *B. cinerea*, Zn cluster TFs BcBOA13 and BcBOT6 are involved in the biosynthesis of important secondary metabolites that function as virulence factors, botcinic acid (BOA) and botrydial (BOT) [90], [91]. Also, BcSMR1 and BcZTF1/2, Zinc cluster TFs, participate in sclerotial melanin biosynthesis [92], [93]. The fungal-specific IPR identifiers “Transcription factor domain, fungi” (IPR007219), and “Fungal transcription factor” (IPR021858) also represent a relevant part of the identified domains (27% in *B. cinerea* and 37.5% in *T. atroviride*), as well as the “Zinc finger C2H2-type” domain (IPR013087), present in all eukaryotes (Table 1; Fig. 2A).

**Table 1:** Transcription factor protein family classification based on InterPro identifiers.

InterPro ID	DBD family names (InterPro)	*B. cinerea*	% of DBD	*T. atroviride*	% of DBD
IPR001138	Zn_Cluster	236	38.2	294	39.1
IPR007219	Transcription factor domain, fungi	107	17.3	196	26.1
IPR021858	Fungal transcription factor	60	9.7	86	11.4
IPR013087	Zinc finger C2H2-type	89	14.4	71	9.4
IPR004827	bZip	22	3.6	24	3.2
IPR009057	Homeobox-like domain superfamily	23	3.7	20	2.7
IPR011598	Myc-type, basic helix-loop-helix (bHLH) domain	9	1.5	10	1.3
IPR009071	High mobility group box domain	9	1.5	7	0.9
IPR000571	Zinc finger, CCCH-type	8	1.3	6	0.8
IPR000679	Zinc finger, GATA-type	7	1.1	6	0.8
IPR001878	Zinc finger, CCHC-type	9	1.5	5	0.7
IPR003163	Transcription regulator HTH, APSES-type DNA-binding domain	4	0.6	5	0.7
IPR001766	Forkhead	4	0.6	4	0.5
IPR000232	HSF	3	0.5	3	0.4
IPR008967	p53-like transcription factor, DNA-binding	3	0.5	3	0.4
IPR001083	Copper fist DNA-binding	3	0.5	2	0.3
IPR002100	Transcription factor, MADS-box	3	0.5	2	0.3
IPR000818	TEA/ATTS domain	1	0.2	1	0.1
IPR001606	ARID DNA-binding domain	3	0.5	1	0.1
IPR003120	Transcription factor Ste12	1	0.2	1	0.1
IPR003150	DNA-binding RFX-type winged-helix domain	2	0.3	1	0.1
IPR003656	Zinc finger, BED-type	1	0.2	1	0.1
IPR007396	Transcriptional regulator PAI 2-type	2	0.3	1	0.1
IPR007604	CP2 transcription factor	1	0.2	1	0.1
IPR017956	AT hook, DNA-binding motif	2	0.3	1	0.1
IPR000967	Zinc finger, NF-X1-type	1	0.2	0	0.0
IPR004181	Zinc finger, MIZ-type	1	0.2	0	0.0
IPR004198	Zinc finger, C5HC2-type	1	0.2	0	0.0
IPR005172	CRC domain	1	0.2	0	0.0
IPR006856	Mating-type protein MAT alpha 1, HMG-box	1	0.2	0	0.0
	**Total**	**617**	**100**	**752**	**100**

The remaining identified domains are typically found in fungal genomes [55], [85] and include DBDs shared with other eukaryotes, such as bZIP, Homeobox, bHLH, and GATA domains, or fungal-specific DBDs, such as APSES, Copper-Fist, Ste12, or MAT alpha 1 (Table 1). Unlike the Zn cluster-containing domain, these fungal-specific domains are less represented (3% or less of the DBDs found). However, they play important roles in fungal development, including mating, morphogenesis, yeast-hyphal transitions, and cell cycle [94], [95], [96], [97], [98], [99], [100], [101].

As expected, a portion of the TFs displayed two DBDs (approximately 2–3% of the TFs in both fungi) (Table 2, Fig. 2B). The most represented combinations found were “Zinc finger C2H2-type”/“Zn Cluster” and the “Zinc finger C2H2-type”/“Homeobox-like domain superfamily” families of TFs (Fig. 2B). The proportion of TFs with two DBDs (also termed “dual-specificity TFs”) is in agreement with that previously reported for other fungi (1–4% of the TFs, [55]).

**Table 2:** Combination of dual DBDs transcription factors.

InterPro DBD Combination (IDs)	Name	*T. atroviride*	*B. cinerea*
IPR001138-IPR013087	Zinc finger C2H2-type/Zn_Cluster	3	7
IPR001606-IPR004198	ARID DNA-binding domain/Zinc finger, C5HC2-type	0	1
IPR001606-IPR009057	ARID DNA-binding domain/Homeobox-like domain superfamily	1	1
IPR001606-IPR003150	ARID DNA-binding domain/DNA-binding RFX-type winged-helix domain	0	1
IPR009057-IPR009071	Homeobox-like domain superfamily/High mobility group box domain	0	1
IPR009057-IPR013087	Zinc finger C2H2-type/Homeobox-like domain superfamily	4	2
IPR013087-IPR017956	Zinc finger C2H2-type/AT hook, DNA-binding motif	0	1
IPR003656-IPR013087	Zinc finger C2H2-type/Zinc finger, BED-type	1	1
IPR003120-IPR013087	Zinc finger C2H2-type/Transcription factor Ste12	1	1
	**TF with 2 DBD Domains**	**10**	**16**

With the defined repertoire of TFs for each fungus, we established the DNA binding preference for each of them. The CisBP database [4] is the most complete source of DNA sequence binding preferences for eukaryotic TFs, comprising 734 species from which 310 correspond to fungi. However, most of the fungal DNA binding motifs in CisBP obtained by direct experimental determination correspond to TFs of the model fungi *N. crassa*, *A. nidulans*, and *S. cerevisiae.* For *B. cinerea*, only one TF, Bcin05g07400, has a directly determined binding preference, and for *T. atroviride*, no direct motifs are available*.* Nevertheless*,* a total of 106 and 104 TFs for *B. cinerea* and *T. atroviride,* respectively*,* have an automatically inferred motif derived from homologous TFs, as the DNA binding specificity can be inferred following general rules that depend on each TF family and the degree of similarity among DBDs. Since most TFs from our curated catalog do not possess any automatically inferred DNA binding motif, we assigned a motif for these TFs using the “Protein Scan” web tool from CisBP or by performing an orthogroup classification as detailed in Methods (Fig. 1A). We were able to assign a putative DNA binding motif for 375 (79.6%) and 423 (75.4%) of the predicted TFs from *B. cinerea* and *T. atroviride,* respectively (Supplementary File 5). In the case of dual-specificity TFs, we assigned two different PWMs (one to each DBD) for four out of 16 TFs in *B. cinerea* and eight out of ten TFs in the biocontroller fungus (Supplementary File 4).

### 3.2 Generation of reference GRNs for *B. cinerea* and *T. atroviride*

To identify TFs target genes, we inferred regulatory interactions based on the distance between a given TFBS and the transcription start site (TSS) of each annotated gene of *B. cinerea* or *T. atroviride*, using FIMO, considering a distance threshold of 1 Kb between the TFBS and the TSS. We found 370*B. cinerea* TFs and 420 *T. atroviride* TFs (represented by 232 and 251 unique PWMs, respectively) having at least one binding site in promoters. The network of TF-targets predicted with FIMO comprises 606,188 putative TF-target interactions for *B. cinerea* and 891,966 potential TF-target interactions in the case of *T. atroviride* (Table 3). To improve FIMO predictions with experimentally derived data, we used GENIE3 (Gene Network Inference with Ensemble of trees 3) [9], a tree-based machine-learning algorithm to infer regulatory interactions from gene expression data. GENIE3 has been widely used to derive GRNs in various species (e.g.[102], [103], [104], [105], [106]) and was the highest-scoring algorithm in the Dialogue for Reverse Engineering Assessments and Methods (DREAM) challenges 4 and 5 [107]. GENIE3 generates a rank of regulatory connections, ranging from the most confident to the least confident interaction. These are available as Supplementary Files 6 and 7. We conducted an exhaustive search for publicly available RNA-Seq data for both fungi from the NCBI SRA. After filtering files for mean quality and a minimum number of mapped reads to both genomes (see Methods), we obtained a final dataset of 228 samples for *B. cinerea* and 163 samples for *T. atroviride* (Supplementary File 8). Samples were processed as described in Methods and expression matrices, as well as the list of TFs for both fungi, were given as input for GENIE3. The GENIE3-generated network comprised 5,516,823 gene interactions for *B. cinerea* and 6,603,528 gene interactions in the case of *T. atroviride*. To remove unlikely interactions, we constrained the network to the top 20% better scoring GENIE3 TF-target interactions. This represents approximately the top one million interactions, a cutoff that has been previously used for filtering GENIE3-generated GRNs [108], [109], [106]. We used this information to filter FIMO-derived predictions, obtaining reference GRNs for both fungi. The FIMO network and the GENIE3-filtered reference network have a similar number of nodes. However, the number of regulatory connections (edges) observed in the FIMO GRN substantially decreased by incorporating expression data in the case of *B. cinerea* (by 3.8-fold) and *Trichoderma* (by 4.9-fold) (Table 3). The reference networks include all (100%) reported *B. cinerea* and *T. atroviride* TFs, indicating that they include relevant information for transcriptional regulatory inferences for both organisms. All networks are available as Supplementary Files 9 to 12 (Cytoscape .cys format).

**Table 3:** Gene regulatory networks statistics. The FIMO-only GRN, as well as the reference (FIMO + GENIE3) GRN, are described.

	FIMO GRN		Reference GRN			
Attribute	*B. cinerea*	*T. atroviride*	*B. cinerea*	TF Description	*T. atroviride*	TF Description
Total number of nodes	11,706	11,771	11,700	-	11,700	-
Total number of interactions	6,06,188	8,91,966	1,58,374	-	1,81,571	-
Average outdegree	1,638.35	2,123.72	436.29	-	432.31	-
Average indegree	51.78	75.77	13.53	-	15.51	-
Maximum outdegree (#); gene ID	6,256; Bcin06g06480	5,049; TRIATDRAFT_10744	3,340; Bcin01g08840	-	1,686; TRIATDRAFT_140885	-
Maximum indegree (#); gene ID	130; Bcin08g00980	175; TRIATDRAFT_300681	60; Bcin07g05430	-	62; TRIATDRAFT_55202	-
Connected components	1	1	1	-	1	-
Clustering coefficient	0.153	0.216	0.082	-	0.083	-
Top 10 hubs for each network; outdegree	Bcin06g06480; 6256	TRIATDRAFT_10744; 5049	Bcin01g08840; 3340	High mobility group box domain TF	TRIATDRAFT_140885; 1686	Transcription factor SFP1
	Bcin13g04090; 5315	TRIATDRAFT_173784; 4708	Bcin13g05200; 3017	Copper fist domain TF	TRIATDRAFT_234627; 1579	C2H2 domain TF
	Bcin10g05560; 5170	TRIATDRAFT_150201; 4555	Bcin11g02190; 2983	Bchox3 Homeobox domain TF	TRIATDRAFT_322580; 1543	Zn_Cluster & TF domain Fungi
	Bcin03g00710; 4497	TRIATDRAFT_78054; 4488	Bcin13g03910; 2153	Zn_Cluster & C2H2 domain TF	TRIATDRAFT_173231; 1442	C2H2 domain TF
	Bcin05g04650; 3609	TRIATDRAFT_173231; 4258	Bcin01g10720; 1977	bZIP TF	TRIATDRAFT_173784; 1393	Homeobox domain and Zinc finger C2H2-type
	Bcin01g08840; 3444	TRIATDRAFT_322580; 4255	Bcin07g06470; 1961	Zn_Cluster & TF domain Fungi	TRIATDRAFT_31689; 1323	Zinc Cluster and fungal specific TF domain
	Bcin12g03330; 3295	TRIATDRAFT_288678; 4128	Bcin13g00670; 1934	Zn_Cluster & TF domain Fungi	TRIATDRAFT_42504; 1305	Zn_Cluster domain TF
	Bcin13g05200; 3033	TRIATDRAFT_163506; 4099	Bcin02g08650; 1892	Bcskn7 Response regulator TF	TRIATDRAFT_22050; 1301	Zn_Cluster & TF domain Fungi
	Bcin08g00680; 3002	TRIATDRAFT_283122; 4087	Bcin10g04060; 1841	Bcftf1 Zn_Cluster & TF domain Fungi	TRIATDRAFT_260571; 1300	C2H2 domain TF
	Bcin11g02190; 3048	TRIATDRAFT_295974; 3917	Bcin12g01230; 1801	Zn_Cluster & TF domain Fungi	TRIATDRAFT_288678; 1293	Homeobox domain and Zinc finger C2H2-type

To characterize the topology of the reference GRNs, their properties were analyzed. Both reference networks comprised a single connected component, indicating that there is a sequence of nodes and edges (path) connecting each node in the network. However, the average clustering coefficient of the networks is low (0.082 in *B. cinerea* and 0.083 in *T. atroviride*). In fact, a relevant proportion of nodes have a clustering coefficient of 0 (see Supplementary Files 9 and 11), indicating neighborhoods of nodes are sparsely connected. Low clustering coefficients have also been reported for other large-scale fungal GRNs [23], [24].

For the phytopathogen, the reference network comprises 11,700 nodes and 158,374 edges. The Botrytis network’s top 10 connected nodes (hubs) correspond to TFs from different families (Table 3), with the most connected node corresponding to Bcin01g08840, an HMG box TF. This TF presents 3340 targets, corresponding to approximately 29% of the genes in the *B. cinerea* network. HMG box-containing TFs are conserved among eukaryotes, and in fungi, they have important roles in mating [110]. However, no reported function for Bcin01g08840 is currently available. Moreover, from the top 10 hubs, only Bcin02g08650 (*bcskn7*) has been characterized as a homolog to the Skn7 response regulator of *S. cerevisiae*, a stress-responsive TF involved in the oxidative stress response, cell cycle, and cell wall biosynthesis and highly conserved among fungi [111]. BcSKN7 is involved in conidiation and sclerotial formation and oxidative and ionic osmotic stress [112]. On the other hand, the gene with the highest indegree was Bcin07g05430. Harboring a cytochrome P450 domain, its function is unknown. Bcin07g05430 is predicted to be controlled by 60 TFs of the *B. cinerea* network.

In the case of *Trichoderma*, the network was comprised of 11,700 nodes and 181,571 interactions (Table 3). The most connected TF was TRIATDRAFT_140885, annotated as “transcription factor SFP1” by the BLAST2GO analysis. In yeast, split-finger protein 1 (SFP1) is a stress and nutrient-responsive TF [113] controlling cell division and growth by directly regulating the transcription of genes required for ribosome biogenesis and growth [114]. As for *B. cinerea*, no direct functional information is available for the top 10 hubs. However, the 13th most connected TF, TRIATDRAFT_83090 (see Supplementary File 11), annotated as a C2H2 domain TF harbors a 90.5% identity with ACEI, a TF encoded by the *ace1* gene in *Trichoderma reesei* [115]*.* ACEI can bind the promoter of the major cellulase cellobiohydrolase I (*cbh1*) gene *in vitro* and *in vivo*. Consistently, disruption of *ace1* in *T. reesei* alters its growth in cellulose medium [115]. The hypothetical protein TRIATDRAFT_55202 has the highest indegree in the network, with 62 regulatory connections.

To further test the generated reference GRNs, and to determine how these networks allow for the prediction of potential TF-target interactions that can result in changes in gene expression, we gathered global gene expression information from different TF mutants in *B. cinerea* and *T. atroviride*, in comparison with their wild-type counterparts [75], [76], [77], [78], [74]. We compared lists of DEGs in wild-type versus TF mutant fungi and lists of potential targets for each TF predicted by the reference GRNs. As shown in Supplementary Table 2, we were able to find a significant overlap between DEGs and predicted targets for four out of six TFs tested. This result indicates that our reference networks can pinpoint relevant TF-target relationships, highlighting their potential to uncover relevant regulatory functions for uncharacterized TFs.

### 3.3 Generation and analysis of GRNs during growth under constant light conditions

Having a reference GRN allows for constructing context-specific networks by mapping regulatory interactions occurring under particular experimental conditions. In fungi, light is considered a strong cue that impacts several biological processes, such as asexual and sexual developmental programs, secondary metabolism, pathogenicity, and even nutrient acquisition [50]. By direct transcriptional control and/or additional signaling pathways involving kinases, light regulates the expression of hundreds of genes. In this context, the transcriptional effects generated by light are considered fundamental, as they trigger transcriptional cascades based on the activation of several TFs [116]. In different fungal organisms, including well-established fungal photobiological models such as *N. crassa* and *A. nidulans*, and other relevant models including *B. cinerea* and *T. atroviride*, the activation of asexual developmental programs is induced by light [117], [118], [119], [120]. In *B. cinerea*, light regulates the production of reproductive structures and virulence [64], restricting sexual reproduction to darkness. In this fungus, different light TFs (termed LTFs) have been studied (reviewed in [120]), and among them, BcLTF2 has been described as necessary and sufficient for conidiation [121]. Photoinduced conidiation has also been described in *T. atroviride*. In this biocontroller fungus, the blue light (transcriptional) regulators 1 and 2 (BLR1 and BLR2) [122], homologs of the White-Collar (transcriptional) Complex (WCC) originally described in *N. crassa* [123] are required for light signaling. Nevertheless, in *T. atroviride*, light transcriptional signaling has been much less investigated [80]*.* Moreover, although some TFs involved in light signaling have been reported in both fungi, a more integrated picture of the regulatory interactions shaping fungal responses to light is still lacking. To determine the GRNs underlying light-dependent gene expression in *B. cinerea* and *T. atroviride*, we generated light-specific GRNs using available transcriptomics data. For *B. cinerea* and *T. atroviride*, we analyzed previously deposited RNA-Seq data from wild-type hyphae exposed to darkness or continuous light (SRA SRP235144 and SRP069026, respectively).

For *B. cinerea*, we determined a total number of 1610 differentially-expressed genes (DEGs) under constant light. From these, 663 genes (42.2%) were induced by the presence of constant light, whereas 947 were down-regulated (58.8%). For *T. atroviride*, 2286 DEGs were determined. Unlike the phytopathogen, where most genes were down-regulated by constant light, for *T. atroviride*, 1545 genes (67.6%) were more expressed in the mentioned culture condition (Supplementary File 13). *Trichoderma* upregulated genes have enriched GO terms associated with five biological processes: metabolic and oxidation–reduction processes, transmembrane transport, and catabolic process related to chitin, a critical structural component of fungal cell walls (Supplementary Fig. 2A). We determined six GO biological processes among repressed DEGs, with related functional categories associated with redox activities and transmembrane transport (Supplementary Fig. 2B).

In contrast, GO analysis of *B. cinerea* DEGs did not reveal any significantly enriched GO term. To determine regulatory interactions driving these changes in gene expression in both fungi, we built GRNs consisting only of DEGs, using the TF-target information from the reference GRNs. Statistics of these context-specific networks are provided in Supplementary Table 3. Remarkably, of the 1610 DEGs in *B. cinerea*, 57 were TFs, and for 53 of them, it was possible to predict putative target genes, generating a network encompassing 82.5% of DEGs. In the case of *T. atroviride*, we could infer regulatory interactions for 61 out of 68 TFs among DEGs, with a GRN that contains 75.7% of the total number of DEGs.

**Fig. 3:**
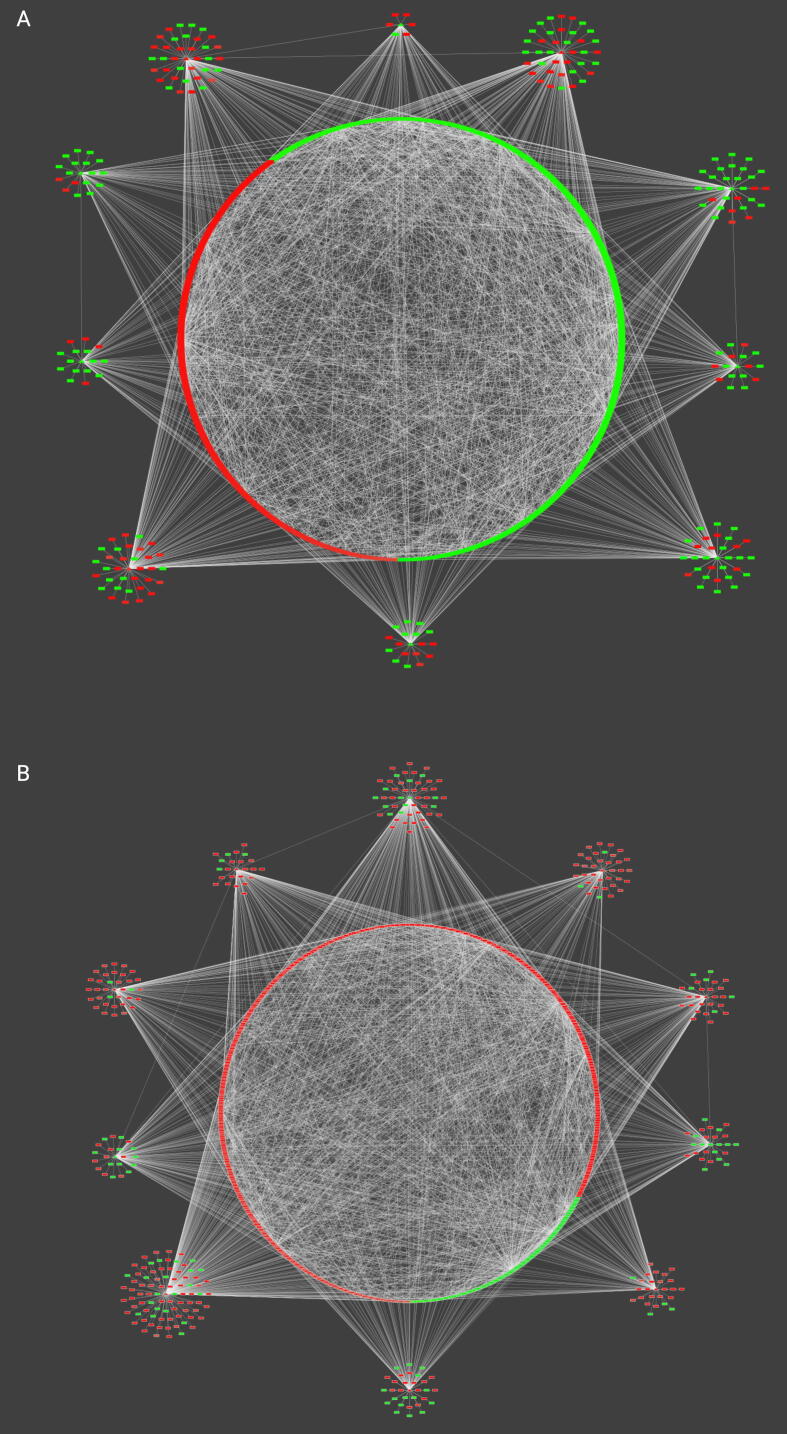
Comparative network analysis of transcriptomics data of *B. cinerea* and *T. atroviride* obtained under continuous illumination*.* Employing publicly available RNA-seq information, *B. cinerea* (A) and *T. atroviride* (B) DEGs determined under constant light conditions were integrated as a GRN of TFs (triangle nodes) and their respective putative target genes (rectangle nodes). Colors are used to distinguish each gene in the network as induced (red) or repressed (green) upon continuous light treatment. Each network was constructed with the reference GRN of *B. cinerea* (A) and *T. atroviride* (B). Both networks only contain the top ten most connected TFs (depicted at the center of each gene module shown in the outer ring). These gene network modules (ten in each case) were grouped by topology in Cytoscape. White edges denote at least a single TFBS in the promoter of each target gene. (For interpretation of the references to colour in this figure legend, the reader is referred to the web version of this article.)

The abovementioned results exemplify the profound transcriptional effect that light has on both organisms, which is beginning to be deciphered in *B. cinerea* [124] but is less explored in *T. atroviride*. To delve into this observation, we paid particular attention to the top 10 most connected TFs for both fungi. These can be found in Table 4 and are represented in the outer ring of Fig. 3A and B. Highlighting a striking difference in the transcriptional landscape of both fungi in the presence of constant light, the majority of the top ten most connected TFs in *B. cinerea* are associated with less expressed (down-regulated) genes in contrast to *Trichoderma*, in which most transcriptional modules (outer ring of Fig. 3B) are regulating more expressed genes during continuous illumination. Importantly, at least in the case of *B. cinerea*, the precise molecular mechanism that may explain this difference is unknown, as light transcription factors (LTFs) are light-induced but not repressed [120]. Possibly reflecting this fundamental difference, none of the most connected TFs in *B. cinerea* is homologous to those in *Trichoderma*, except for the TEA/ATTS domain TFs Bcin05g04650 and TRIATDRAFT_322845, which show very low sequence identity between them (Table 4). Bcin05g04650 is similar to the *A. nidulans* AbaA regulator, a TEA-ATTS domain TF that regulates the development of conidiophores in this fungus [125], but its role in the development of these structures in *B. cinerea* has not been analyzed. In *A. nidulans*, the conidiation process has been extensively studied, describing a sequential cascade of TF activation formed by BlrA, AbaA, and WetA [126]. Consistent with a common transcriptional regulatory cascade occurring in *B. cinerea*, the third most connected TF in the phytopathogen GRN is BcLTF2 (Bcin16g02090), a key regulator of photomorphogenesis and functional counterpart of BlrA [120].

**Table 4:** Top ten most connected transcription factors in the light-specific GRNs of *B. cinerea* and *T. atroviride*. The outdegree (number of targets) of each indicated transcription factor is provided.

*B. cinerea* TF ID	Number of targets	Name	Description	Best BLASTp hit in *Trichoderma;* e-value; %ID
Bcin02g02300	183	n.a.	SANT/Myb & Homeobox-Like domain TF	TRIATDRAFT_289913; 4.4e-69; 83.7
Bcin02g01550	151	n.a.	Basic-leucine zipper domain TF	TRIATDRAFT_297702; 6.3e-12; 50.0
Bcin16g02090	148	BcLTF2	C2H2 domain TF. Light induced TF	TRIATDRAFT_165197; 5.9e-44; 41.8
Bcin15g05300	139	n.a.	p53-like transcription factor	TRIATDRAFT_225495; 7.6e-17; 74.5
Bcin05g04650	129	BcAbaA	TEA/ATTS domain TF. Similar to *A. nidulans* AbaA TF	TRIATDRAFT_322845; 1.4e-10; 64.1
Bcin02g08760	100	BcSMR1	Zn_Cluster & C2H2 domain TF. Sclerotial Melanin Regulator	TRIATDRAFT_295411; 7.6e-14; 50.9
Bcin14g03200	95	n.a.	Zn_Cluster & Fungal_TF domain TF	TRIATDRAFT_222577; 7.2e-17; 55.9
Bcin11g06200	92	n.a.	C2H2 domain TF	TRIATDRAFT_173231; 1.1e-17; 50.8
Bcin02g09340	91	n.a.	Zn_Cluster & C2H2 domain TF	TRIATDRAFT_314109; 7.1e-17; 46.7
Bcin04g03280	86	n.a.	C2H2 domain TF	TRIATDRAFT_161626; 0.00035; 56.5

***T. atroviride*****TF ID**	**Number of targets**	**Name**	**Description**	**Best BLASTp hit in*****Botrytis*****; e-value; %ID**

TRIATDRAFT_322845	316	n.a.	TEA/ATTS domain TF	Bcin05g04650; 1.3e-10; 64.1
TRIATDRAFT_53983	231	n.a.	C2H2 domain TF	Bcin13g04470; 5e-38; 79.7
TRIATDRAFT_51777	154	n.a.	Zn_Cluster domain TF	Bcin06g03110; 1.1e-07; 64.5
TRIATDRAFT_174866	154	n.a.	Zn_Cluster domain TF	Bcin08g00160; 6.3e-19; 36.1
TRIATDRAFT_167723	154	n.a.	C2H2 domain TF	Bcin16g02090; 0.005; 22.7
TRIATDRAFT_266277	151	n.a.	Zn_Cluster domain TF	Bcin08g06990; 1.2e-07; 58.6
TRIATDRAFT_13008	135	n.a.	Zn_Cluster & Fungal_TF domain TF	Bcin12g03530; 3.5e-28; 52.8
TRIATDRAFT_287602	131	n.a.	Zn_Cluster domain TF	Bcin01g11410; 4.1e-08; 39.3
TRIATDRAFT_19824	131	n.a.	HMG domain TF	Bcin09g02870; 1.4e-22; 55.9
TRIATDRAFT_301028	127	n.a.	C2H2 domain TF	Bcin07g06370; 8.5e-40; 96.2

Other conserved and highly connected TF in both GRNs are BcSMR1 (Bcin02g08760) (Table 4) and TRIATDRAFT_311296 (26th most connected TF in *T. atroviride*; see Supplementary File 12). The BcSMR1 TF has been characterized in *B. cinerea* as one of the key regulators of melanin synthesis [93]. Commonly, fungal genes that participate in secondary metabolism are encoded in gene clusters, usually containing one TF responsible for the cluster’s expression [127]. In *B. cinerea*, melanin biosynthetic genes are encoded in two gene clusters while other genes are in non-cluster genomic locations. Two polyketide synthetases (PKS), *bcpks12* and *bcpks13*, are key enzymes for melanin production for sclerotia and conidia, respectively, and are encoded in two different clusters. *bcpks12* is clustered with the *bcsmr1* TF, while *bcpks13* is clustered with the *bcztf1* and *bcztf2* TFs and the *bcscd1* and *bcbrn2* genes [128]. *bcpks12* is expressed during sclerotia development, which occurs in the dark, and its expression has been reported to be controlled by the *bcsmr1* TF [93]. Although *bcpks12* and *bcsmr1* – members of the first cluster of biosynthetic genes – are repressed by light in our analysis and are nodes in the *B. cinerea* light-GRN, no regulatory interaction between these genes was found. However, when we analyzed the gene members of the second gene cluster, we observed that *bcztf2*, *bcbrn2*, and *bcpks13* are more expressed in the presence of constant light, and in this case, the GRN predicts that *bcbrn2* is directly regulated by *bcztf2*. As previously suggested [93], since light induces conidiation and therefore conidia melanogenesis via *bcpks13*, it is expected that some additional yet uncharacterized LTFs may play a role in this process. According to the light-GRN, BcLTF15 may regulate the expression of *bcbrn1* as well as the expression of *bcpks13* (Supplementary Fig. 3), revealing a new potential link between melanogenesis and light that has not been previously experimentally determined.

### 3.4 Grns during the mycoparasitic interaction between *B. cinerea* and *T. atroviride*

In addition to an array of molecular tools that *Trichoderma* possesses to counteract different phytopathogens [129], this fungus displays complex inter/intra-phyla association mechanisms. For example, *Trichoderma* can induce the plant’s activation of complex immune responses when associated with plants. This requires phytohormones and MAP kinases signaling cascades and plant TFs acting as critical regulators of the plant response [130], [131], priming the plant for future pathogen encounters. While transcriptional regulators of these responses have been determined in plants, including WRKYs, MYBs, and MYCs-type plant-TFs [131], much less is known about the transcriptional reprogramming occurring at the fungal level. On the other hand, although somewhat more studied, the *Trichoderma*-fungal interaction scenario is far from being entirely understood. Early efforts employing ESTs sequencing and 454 gene expression analysis contributed evidence of transcriptional changes in *Trichoderma* induced by *B. cinerea* cell-wall derivates [132] or expressed during the interaction with *B. cinerea* [133], and additional genes associated with the mycoparasitsm of *Rhizoctonia solani* [134]. Therefore, more attempts are needed to better depict the transcriptional responses at both sides of the equation: the mycoparasite and the fungal organism under attack.

To gain insights into the transcriptional response of the *Trichoderma*-*Botrytis* interaction and to identify candidate key TFs of this process, we first carried out a confrontation assay (Supplementary Fig. 1) and analyzed the changes in the whole transcriptome of both fungi using RNA-Seq. We found that the interaction between *B. cinerea* and *T. atroviride* elicited a different response regarding the total number of DEGs in each fungus, being significantly stronger in the biocontroller. We determined 283 upregulated genes and 255 downregulated genes in *Trichoderma* during the interaction*,* and only 128 upregulated and five downregulated genes in *B. cinerea* (Supplementary File 14). During the *Trichoderma*-*Botrytis* interaction, enriched GO terms among *Trichoderma* induced genes include carbohydrate metabolic process, oxidative stress, and proteolysis (see below; Supplementary Fig. 4A). Nevertheless, no enriched GO terms were identified among *B. cinerea* DEGs.

**Fig. 4:**
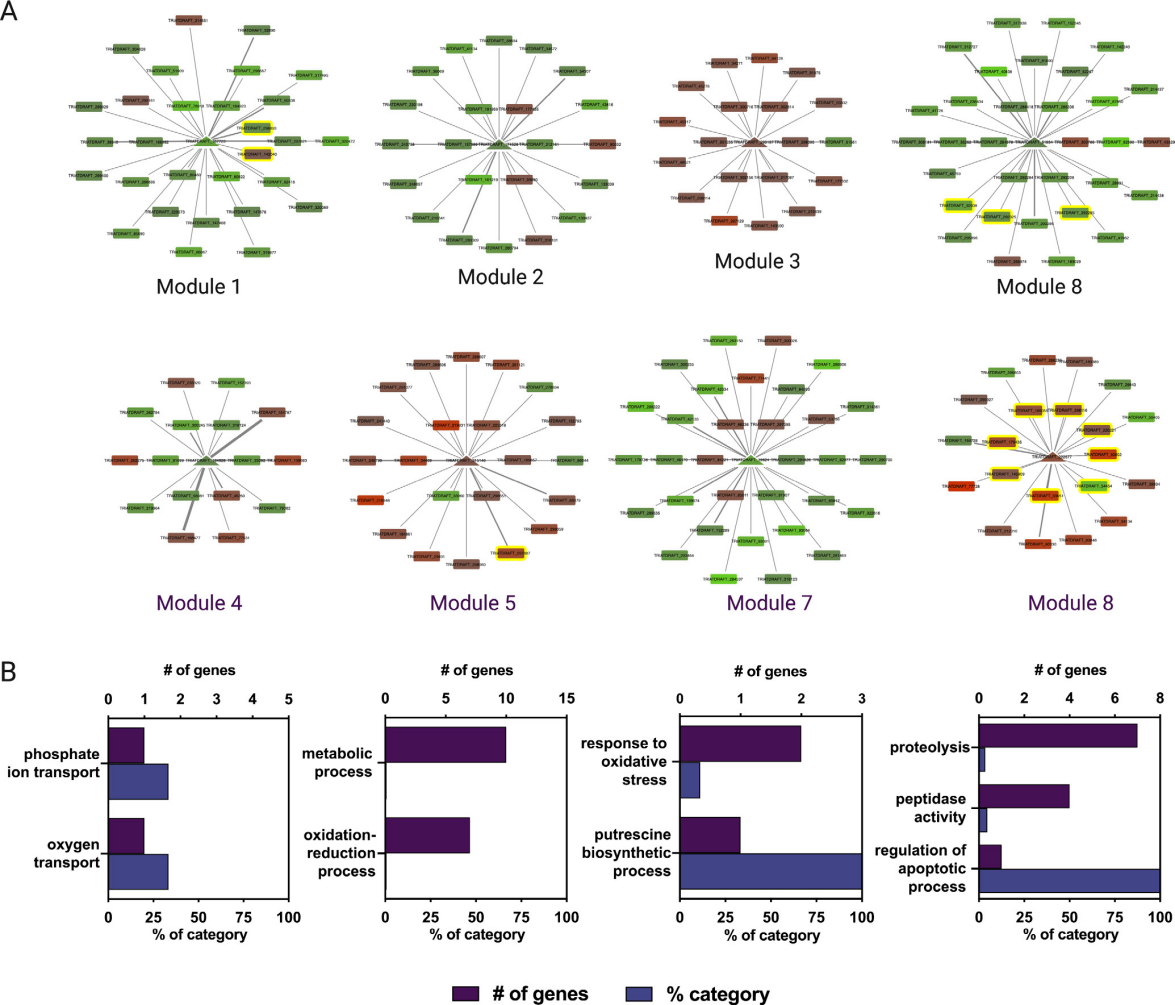
Confrontation-specific GRN pinpoints *T. atroviride*-responsive transcriptional regulators showing differential expression during the interaction with *B. cinerea*. (A) Using the GLay community clustering algorithm in Cytoscape, eight differentially expressed gene modules were identified, each of them regulated by a single TF. Transcriptional regulators are denoted as triangles located at the center of each gene module, while target genes are indicated as rectangles. *T. atroviride* confrontation-induced or repressed genes are shown as red and green nodes, respectively. Gene encoding for peptidases (in modules 1, 5, 6, and 8) are displayed as yellowed-shadowed nodes. Edges denote at least a single TFBS for each target gene. The width of the edges is proportional to the number of TFBS. (B) Overrepresented GO terms (biological processes) are indicated below each gene module shown in (A). While modules 1, 2, 3, and 6 did not deliver any enriched GO term, module 8 is enriched in protein degradation terms consistent with the presence of eight peptidase encoding genes. Bars represent the number of genes observed in each GO category (purple) and their respective percentage (blue). (For interpretation of the references to colour in this figure legend, the reader is referred to the web version of this article.)

To pinpoint key TFs commanding the transcriptional responses of both fungi, we next used each set of DEGs to generate confrontation-GRNs, using the reference GRN described above. In addition, we employed the Community Cluster algorithm [73] of Cytoscape to generate clusters of highly interconnected nodes (referred to as “modules”) to determine groups of genes with common regulators that could be functioning in conjunction to control common biological processes among DEGs. In *T. atroviride*, eight modules of DEGs were observed, representing 37.4% of DEGs (Fig. 4A). Four out of the eight modules were enriched in genes with GO terms related to “phosphate ion transport” (Module 4), “metabolic process” (Module 5), “response to oxidative stress” (Module 7), and “proteolysis/peptidases” (Module 8) (Fig. 4B). Since peptidases represent a potential *T. atroviride* antimicrobial strategy, this latter group of genes was further analyzed. Employing SignalP [135] and DeepLoc software [136], we evaluated the presence of signal peptides and potential extracellular localization signals, respectively, among all DEGs encoding peptidases (Table 5). Twenty-one out of 22 DEGs encoding putative peptidases displayed a predicted signal peptide, with 16 of them having a hypothetical extracellular localization. As observed in Fig. 4A, peptidases were associated with four TFs (TRIATDRAFT_167723, TRIATDRAFT_315146, TRIATDRAFT_51934, and TRIATDRAFT_222577). Among these TFs, TRIATDRAFT_222577 was the most connected predicted regulator of peptidases (Module 8 in Fig. 4A). This TF displays a 76.3% identity with the lscL transcriptional regulator of *Trichoderma asperellum*. The IscL TF was originally described in *Purpureocillium lilacinum* [137], and its overexpression in this fungus was shown to increase the production of leucinostatins [138], lipopeptide antibiotics possessing broad biological activity, including fungi. Although several analogs of these molecules have been identified in a few fungal species, including *P. lilacinum*, there is no evidence of their production in *T. atroviride* [139]. The role of these types of TFs in controlling the expression of these peptidase-encoding genes remains to be experimentally validated.

**Table 5:** Peptidase encoding genes identified among *T. atroviride* differentially expressed genes observed during the mycoparasitic interaction with *B. cinerea*. The table indicates predicted peptidase localization, signal peptide, and the corresponding gene modules depicted in Fig. 4. (“up” and “down”: induced or repressed *T. atroviride* peptidase encoding gene during the interaction with *B. cinerea*).

Gene ID	Description	Signal peptide	SignalP pvalue	Predicted localization	Module in Fig. 4	Expression
TRIATDRAFT_142040	aspartic-type endopeptidase	yes	0.8964	extracellular	1	up
TRIATDRAFT_296893	putative amonio peptidase	no	0.0005	cytoplasm	1	down
TRIATDRAFT_297887	aspartic-type endopeptidase	yes	0.9825	extracellular	5	up
TRIATDRAFT_292285	peptidase	yes	0.0112	ER	6	down
TRIATDRAFT_292325	metallocarboxypeptidase	yes	0.7240	ER	6	down
TRIATDRAFT_32938	aspartic-type endopeptidase	yes	0.9970	membrana	6	down
TRIATDRAFT_50602	serine-type carboxypeptidase	yes	0.0030	lysosome	8	up
TRIATDRAFT_179435	metallopeptidase	yes	0.0122	extracellular	8	up
TRIATDRAFT_33651	aspartic-type endopeptidase	yes	0.7340	extracellular	8	up
TRIATDRAFT_298116	aspartic-type endopeptidase	yes	0.8661	extracellular	8	up
TRIATDRAFT_54454	serine-type endopeptidase	yes	0.9441	extracellular	8	down
TRIATDRAFT_220221	serine-type endopeptidase	yes	0.9603	extracellular	8	up
TRIATDRAFT_145909	serine-type endopeptidase	yes	0.9623	extracellular	8	up
TRIATDRAFT_185055	metallopeptidase	yes	0.9853	extracellular	8	up
TRIATDRAFT_145930	serine-type endopeptidase	yes	0.0007	cytoplasm	DEG, not in modules	up
TRIATDRAFT_292296	aspartic-type endopeptidase	yes	0.8627	extracellular	DEG, not in modules	up
TRIATDRAFT_89596	serine-type endopeptidase	yes	0.8966	extracellular	DEG, not in modules	up
TRIATDRAFT_288190	serine-type endopeptidase	yes	0.9125	extracellular	DEG, not in modules	up
TRIATDRAFT_291825	peptidase	yes	0.9744	extracellular	DEG, not in modules	down
TRIATDRAFT_296905	aspartic-type endopeptidase	yes	0.9792	extracellular	DEG, not in modules	up
TRIATDRAFT_188756	serine-type endopeptidase	yes	0.9885	extracellular	DEG, not in modules	up
TRIATDRAFT_137800	metallocarboxypeptidase	yes	0.9892	extracellular	DEG, not in modules	up

**Fig. 5:**
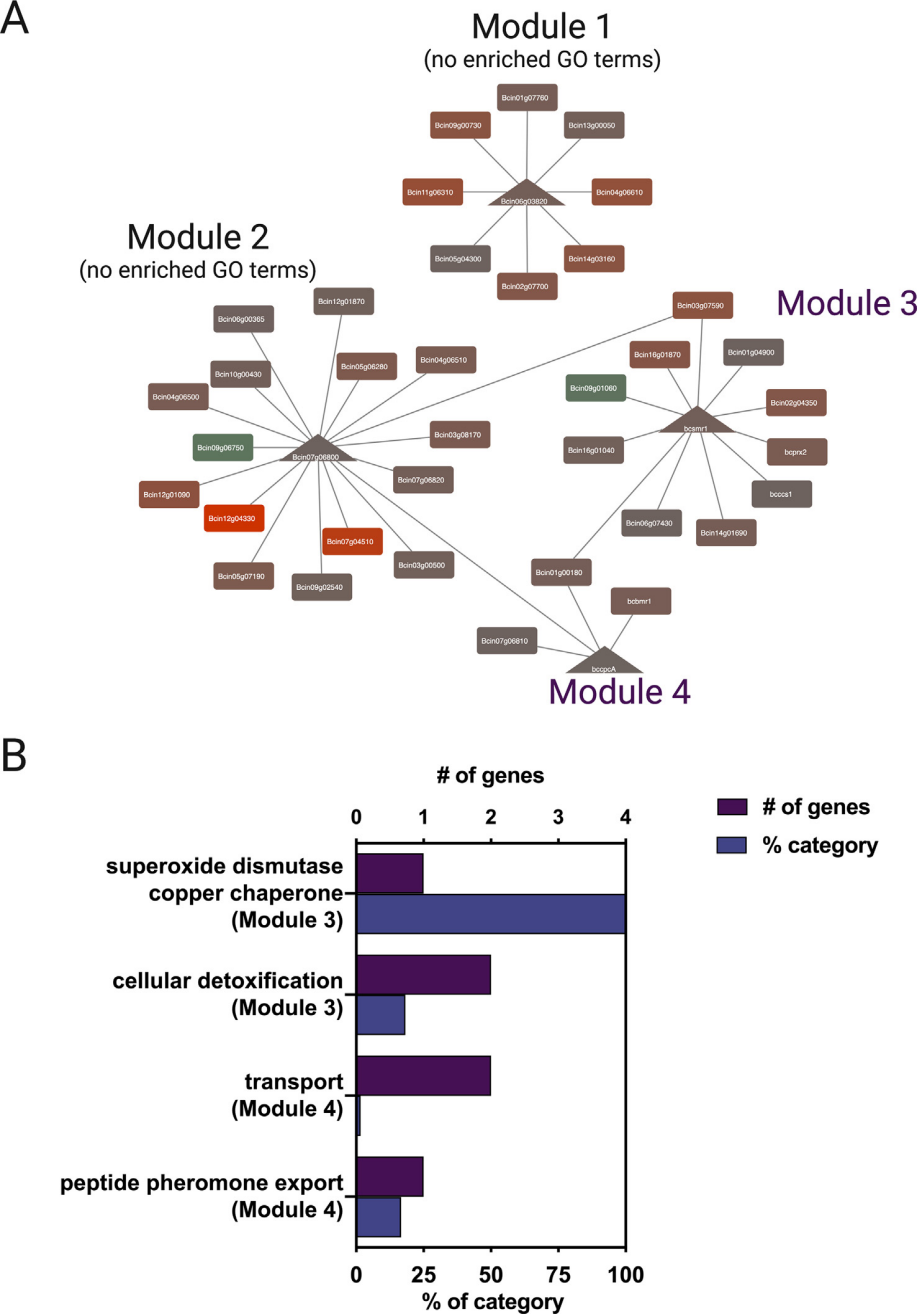
Network analysis of *B. cinerea* transcriptomics reveals gene modules potentially involved in the interaction with the biocontroller fungus *T. atroviride*. (A) *B. cinerea* DEGs determined upon interaction with *T. atroviride* were visualized as a confrontation-specific GRN. Four gene modules were determined after clustering in Cytoscape. Triangles depict TFs, while rectangles denote target genes. *B. cinerea* genes induced or repressed upon interaction with *T. atroviride* are indicated as brown/red and green nodes, respectively. Edges denote at least a single TFBS for each target gene. The width of the edges is proportional to the number of TFBS. (B) Overrepresented GO terms (biological processes) were observed in gene modules 3 and 4 shown in (A). According to the GRN analysis, these modules are controlled by BcSMR1 and BcCPCA TFs, respectively. Purple bars represent the number of genes observed in each GO category, while blue bars indicate their respective percentage. (For interpretation of the references to colour in this figure legend, the reader is referred to the web version of this article.)

In comparison with *Trichoderma*, the analysis of the *B. cinerea* confrontation-GRN was significantly smaller, presenting only four modules of DEGs, each one of them being controlled by a single TF (Fig. 5A). This network comprises mainly upregulated genes representing only 30.1% of the total numbers of DEGs. While modules 1 and 2 did not reveal any enriched GO term, module 3 was associated with protection against oxidative stress (Fig. 5B). In this latter module, confrontation-induced genes in *B. cinerea* include *bcccs1* and *bcprx2*, the former encoding a copper chaperone required for superoxide dismutase function, and the latter encoding a peroxiredoxin. Interestingly, module 3 is controlled by BcSMR1, one of the most connected TFs in the *Botrytis* light-GRN [128]. Melanin, among a myriad of functions [140], protects fungi from extremely harsh environments. Though required for sclerotia melanogenesis in *B. cinerea*, the constitutive expression of *bcsmr1* renders melanin increase [128]. According to the confrontation-GRN, BcSMR1 is predicted to control Bcin02g04350, a hypothetical protein-encoding gene with no reported function that is adjacent (physically linked) to the melanogenic gene BcYGH1 (Bcin02g04360). Finally, GO enrichment analysis of module 4 showed overrepresented terms associated with “export” and “transport”, potentially reflecting *Botrytis*-induced defenses to toxic molecules upon interaction with *T. atroviride*. In this regard, module 4 includes the *B. cinerea* BcBMR1 ABC transporter (Bcin01g05890) [141] and Bcin01g00180, encoding a putative ATP-dependent multidrug transporter (also controlled by BcSMR1). *B. cinerea* mutants deficient in *bcbmr1* are more sensitive to iprobenfos (an organic thiophosphate molecule) and polyoxin [142], both used as agrochemicals. As predicted in the confrontation-GRN, the bZIP TF Bcin01g10810 in Module 4 (Fig. 5A, bottom; annotated as *bccpcA*) might control the expression of these genes. In Aspergillus, *cpcA* encodes a functional orthologue of *S. cerevisiae* Gcn4p TF, and mutants lacking *cpcA* are less virulent [143]. Gcn4p, originally described as critical during amino acid starvation in *S. cerevisiae*, plays a role in the so-called cross-pathway control in *A. nidulans* and *N. crassa* [144], [145], although other regulatory functions have been identified for homologs of this gene, including sexual development [146] and stress responses [147]. DEGs encoding transporters also include *bcatrA* (Bcin11g04460) [148], and Bcin15g00040, a predicted Major Facilitator Superfamily (MFS) transporter. The *bcatrA* gene has a low expression during vegetative growth and is induced during the initial stages of *B. cinerea* infection. When heterologously expressed in *S. cerevisiae*, *bcatrA* confers augmented resistance to cycloheximide and catechol. As suggested, in the absence of defined substrate specificity, it could also protect *B. cinerea* from toxic compounds during saprophytic growth [148] in contrast to *bcatrB* that protects *B. cinerea* against the phytoalexin camalexin during the infection of *A. thaliana* plants [149].

## 4 Conclusions

In aggregate, our work provides a highly valuable resource of regulatory interactions in *B. cinerea* and *T. atroviride*, including a curated set of TFs for both fungi, based on their latest annotation. We show with two different examples that the reference networks can be integrated with global gene expression data to guide the development of context specific GRNs. The light and interaction networks offer novel hypotheses of transcriptional control of relevant biological processes, including attack and defense strategies in *T. atroviride* and *B. cinerea*, respectively, pinpointing key TFs that can be further experimentally validated.

## Funding

This work was supported by ANID – Millennium Science Initiative Program - ICN17_022 to L.F.L., E.A.V., and P.C., ANID-PCI REDI170218 to C.O.Y. and REDES180097 to E.A.V., ANID FONDECYT POSTDOCTORADO N° 3190628 to C.O.Y., ANID FONDECYT POSTDOCTORADO N° 3180328 TO A.S., FONDECYT-Regular 1190611 to P.C., International Research Scholar program of the Howard Hughes Medical Institute to L.F.L. Powered@NLHPC: This research was partially supported by the supercomputing infrastructure of the NLHPC (ECM-02).

## CRediT authorship contribution statement

**Consuelo Olivares-Yañez:** Conceptualization, Investigation, Methodology, Data curation, Formal analysis, Validation, Visualization, Writing – original draft, Writing – review & editing. **Evelyn Sánchez:** Investigation, Methodology, Data curation, Formal analysis, Writing – review & editing. **Gabriel Pérez-Lara:** Investigation, Methodology, Data curation, Formal analysis, Writing – review & editing. **Aldo Seguel:** Formal analysis, Data curation, Writing – review & editing. **Pamela Y. Camejo:** Investigation, Methodology, Formal analysis, Writing – review & editing. **Luis F. Larrondo:** Resources, Funding acquisition, Conceptualization, Writing – review & editing. **Elena A. Vidal:** Supervision, Resources, Funding acquisition, Project administration, Conceptualization, Data curation, Formal analysis, Validation, Visualization, Writing – original draft, Writing – review & editing. **Paulo Canessa:** Supervision, Resources, Funding acquisition, Project administration, Conceptualization, Data curation, Formal analysis, Validation, Visualization, Writing – original draft, Writing – review & editing.

## Declaration of Competing Interest

The authors declare that they have no known competing financial interests or personal relationships that could have appeared to influence the work reported in this paper.
