# Supplementary figures and images for: A comprehensive transcription factor and DNA-binding motif resource for the construction of gene regulatory networks in Botrytis cinerea and Trichoderma atroviride
Source: Comput Struct Biotechnol J. 2021 Nov 18;19:6212–28. doi: 10.1016/j.csbj.2021.11.012 (PMC8637145; doi:10.1016/j.csbj.2021.11.012)

## Slide 1
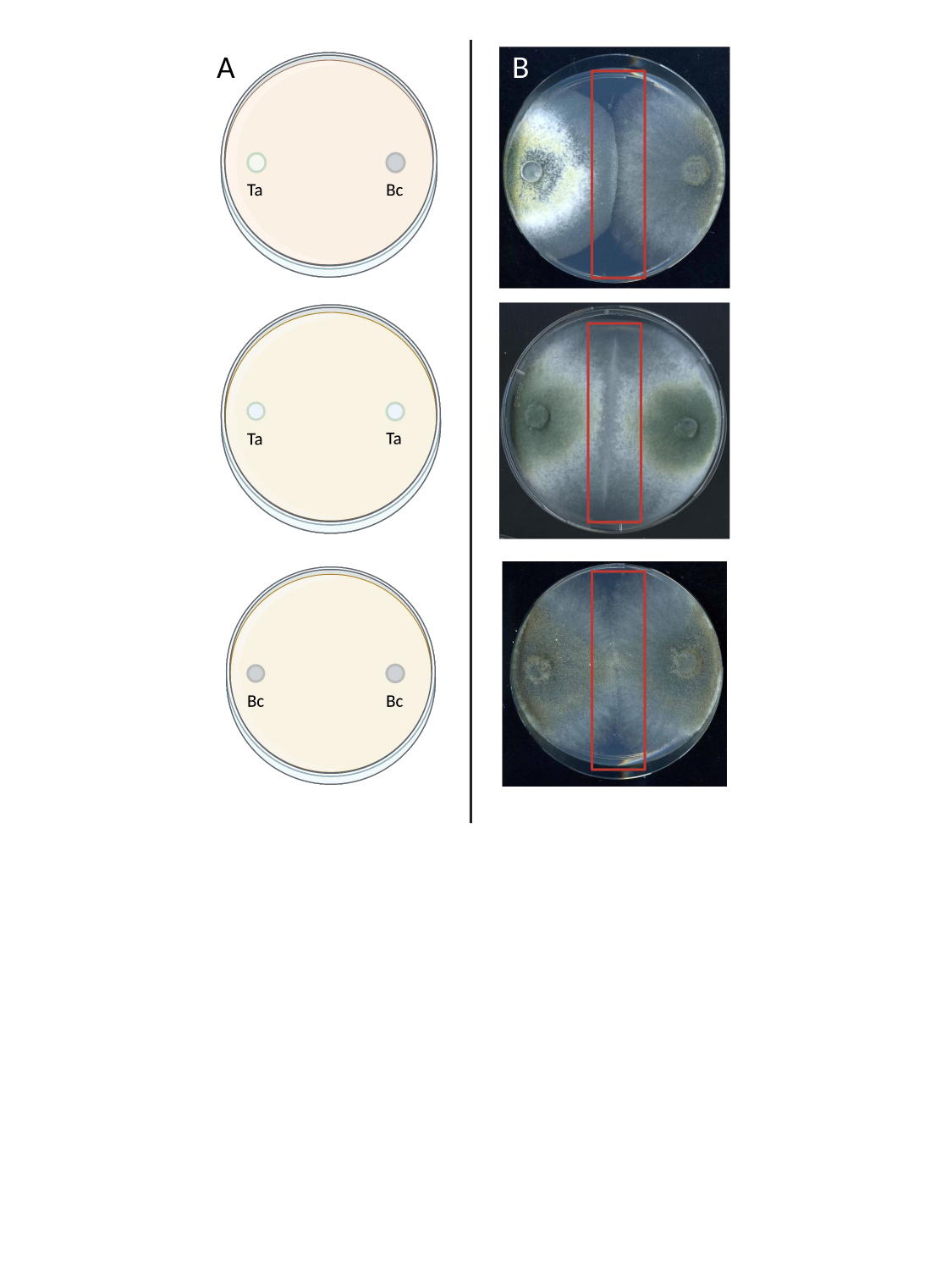

A
B

Supplement: Supplementary data 1 [file mmc1.pptx]

## Slide 1
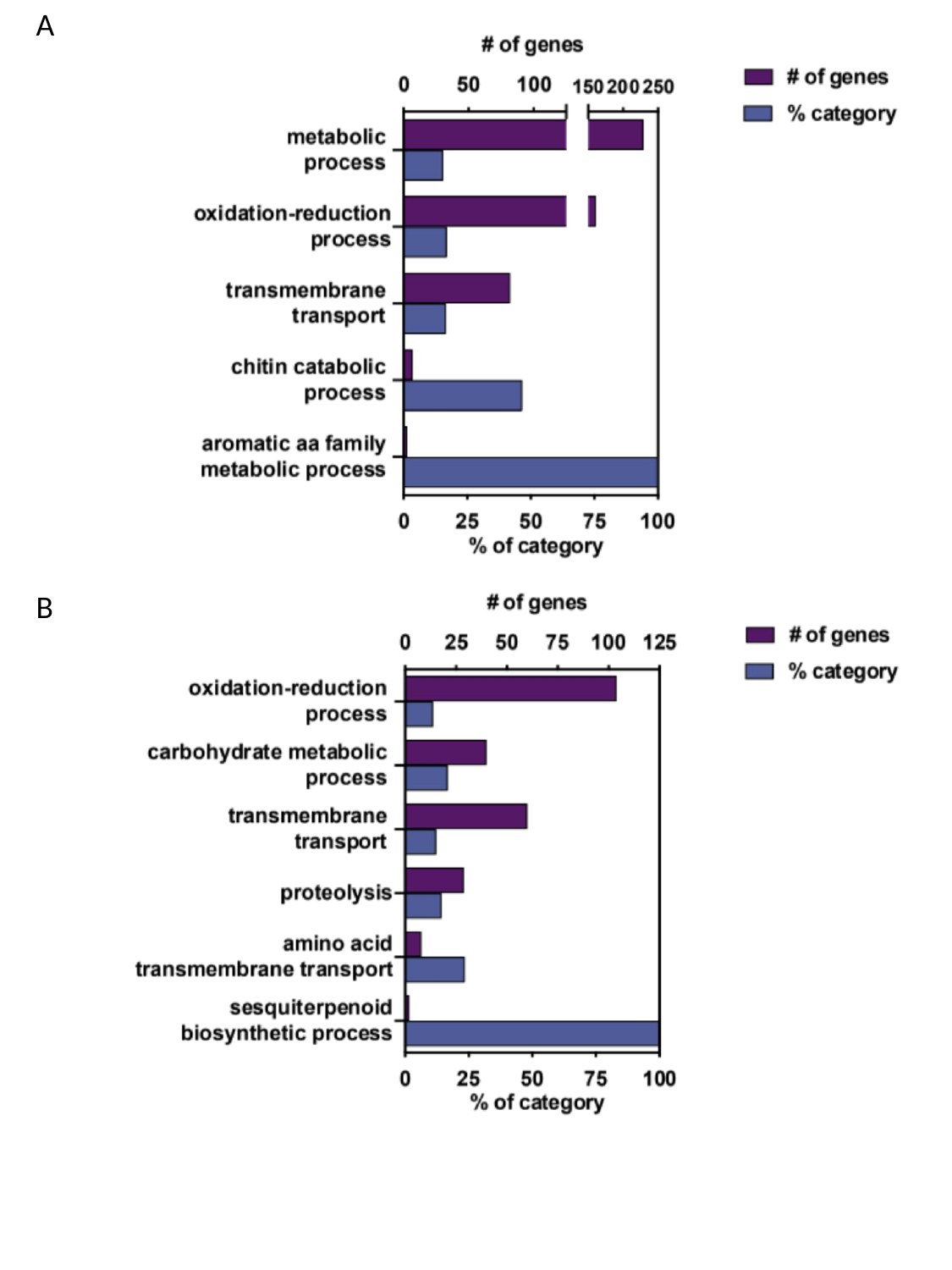

A
B

Supplement: Supplementary data 2 [file mmc2.pptx]

## Slide 1
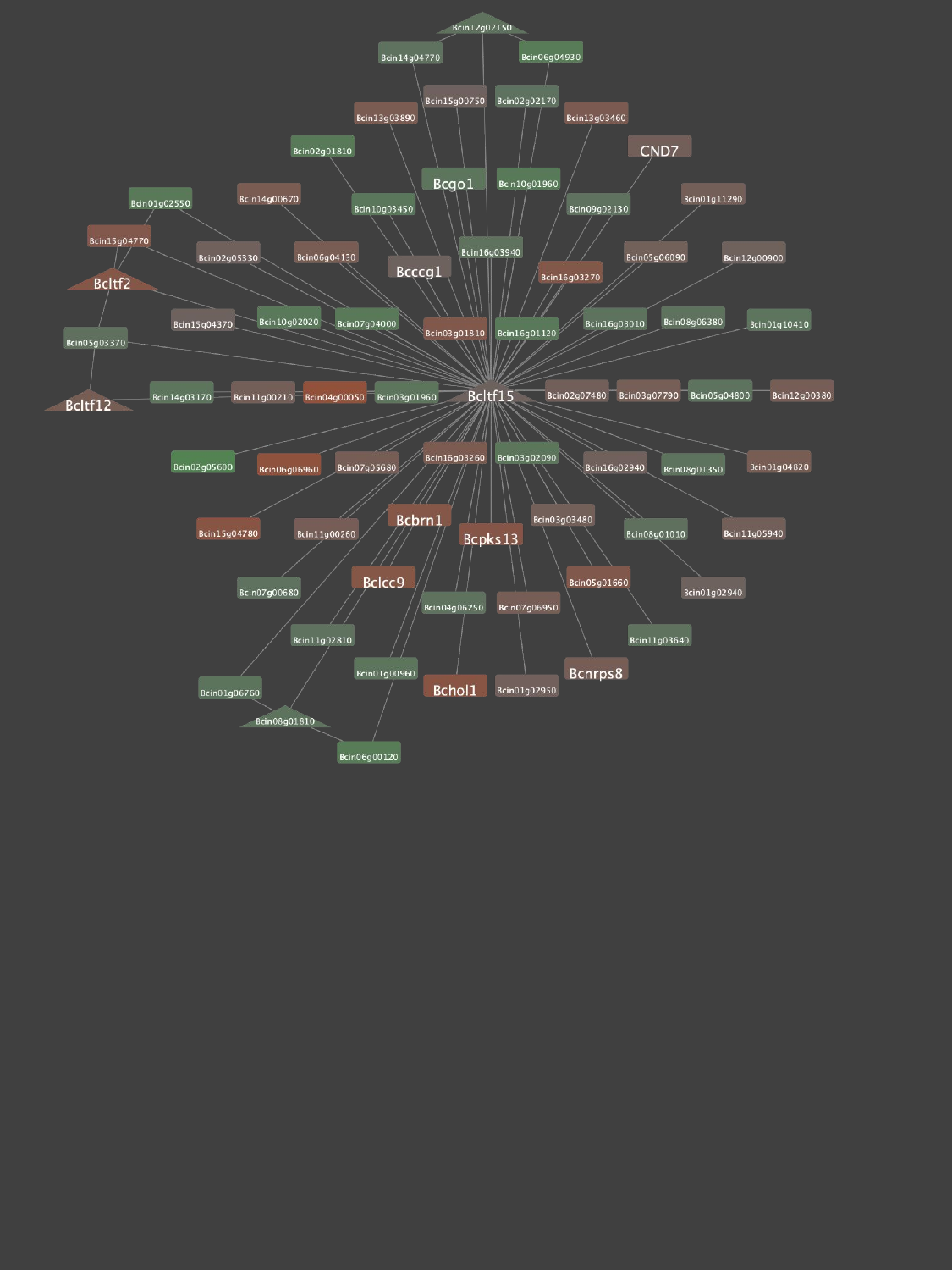

Supplement: Supplementary data 3 [file mmc3.pptx]

## Slide 1
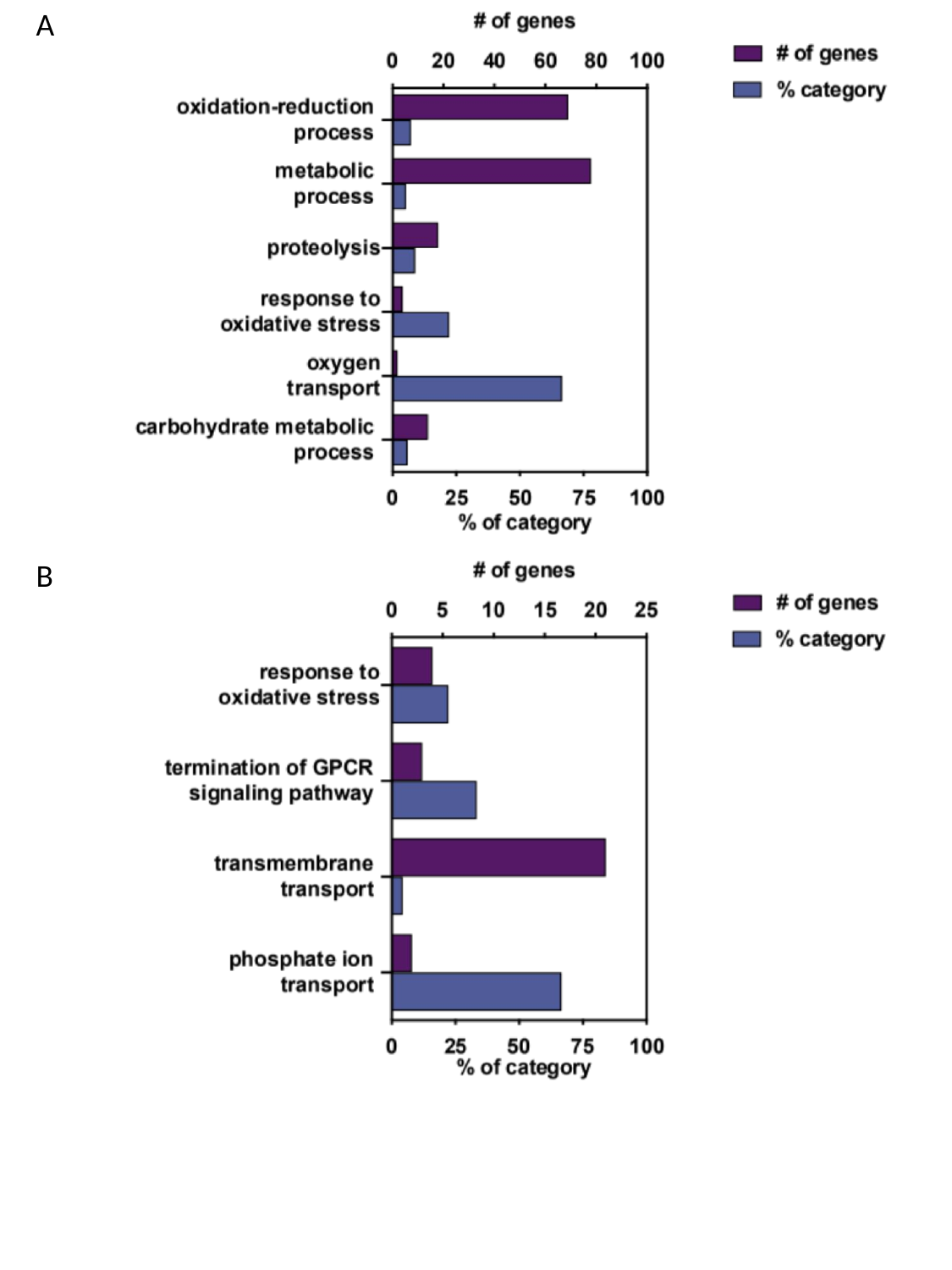

A
B

Supplement: Supplementary data 4 [file mmc4.pptx]
