# Supplementary material for: A comprehensive transcription factor and DNA-binding motif resource for the construction of gene regulatory networks in Botrytis cinerea and Trichoderma atroviride
Source: Comput Struct Biotechnol J. 2021 Nov 18;19:6212–28. doi: 10.1016/j.csbj.2021.11.012 (PMC8637145; doi:10.1016/j.csbj.2021.11.012)
Supplement: Supplementary data 22 [file mmc22.docx]

# Supplementary material.

**Supplementary Fig. 1. *B. cinerea-T. atroviride* on-plate dual confrontation assay. (A)**Schematic representation of the fungal confrontation assay performed on Petri dishes containing PDA culture media. As shown in the diagram, a mycelium agar plug of each fungus (Bc, *B. cinerea*, Ta, *T. atroviride*) was placed on opposite sides of a Petri dish (top panel), allowing fungi to grow. As depicted, control plates (middle and bottom panels) were inoculated only with *B. cinerea* or *T. atroviride.* **(B)** Representative image of the confrontation assay (top panel) after 3 days post-inoculation. In each plate, the red rectangle indicates the area of tissue harvesting, further processed for RNA extraction and sequencing, as indicated in Methods.

**Supplementary Fig. 2. Bar charts depicting enriched GO terms observed among *T. atroviride*induced (A) and down-regulated (B) genes in the presence of constant light.** Only GO terms in the biological process category, and the number of genes belonging to each GO term (purple) are shown. Blue bars represent the percentage observed in each GO category.

**Supplementary Fig. 3. Network analysis of the *B. cinerea* gene module regulated by the BcLTF15 TF under continuous illumination*.*** Employing publicly available RNA-seq information, *B. cinerea* DEGs determined under constant light conditions were integrated as a GRN of the TF BcLTF15. TFs (triangle nodes) and their respective putative target genes (rectangle nodes) are depicted in the figure. Colors are used to distinguish each gene in the network as more (red) or less (green) expressed upon continuous light treatment. The network was constructed with the reference GRN of *B. cinerea.*White edges denote at least a single TFBS in the promoter of each target gene.

**Supplementary Fig. 4. GO term enrichment analysis of differentially expressed *T. atroviride* genes during the mycoparasitic interaction with *B. cinerea*.**Bar charts depicting enriched GO terms observed among confrontation-induced **(A)** and confrontation-repressed **(B)** differentially expressed genes in *T. atroviride*during the interaction with *B. cinerea*. Only GO terms in the biological process category are shown. Purple bars depict the number of genes belonging to each GO term, while blue bars represent the percentage observed in each GO category.

**Supplementary Table 1. PFAM identifiers used for transcription factor identification and further manual curation and selection.**Complete list of PFAM IDs used to identify putative transcription factors in the proteome of *B. cinerea* and *T. atroviride*.

**Supplementary Table 2. Gene overlap between predicted targets of each indicated TF and differentially expressed genes determined in their respective loss-of-function mutant strain.** The statistical significance of the intersection between “Predicted TF targets” and “DEGs in TF mutant” was calculated with the GeneOverlap R package as indicated in Methods (p<0.05).

**Supplementary Table 3. Basic statistics of context-specific gene regulatory networks of *B. cinerea*and*T. atroviride.***Statistics of both light and confrontation-specific GRNs are provided*.*

**Supplementary File 1. InterPro identifiers used for transcription factor identification and further manual curation and selection.**Complete list of InterPro IDs used to identify putative transcription factors in the proteome of *B. cinerea* and *T. atroviride*.

**Supplementary File 2. HMM, profiles employed for transcription factor identification.**The table contains the complete list of HMM profiles used to identify putative transcription factors in the proteome of *B. cinerea* and *T. atroviride*.

**Supplementary File 3. Annotations and criteria employed for transcription factors manual curation in *B. cinerea* and*T. atroviride*.**All protein sequences were retrieved and analyzed after the InterPro and HMM queries indicated in Fig. 1. The table provides BLAST2GO and FungiFun protein functional descriptions, as well as gene names (when available) and the manual curation of each TF. The TF definition for *B. cinerea* and *T. atroviride* is indicated in each table’s fourth and third columns, respectively.

**Supplementary File 4. Dual-specificity TFs identified in *B. cinerea*and *T. atroviride*and their corresponding Position Weight Matrix (PWM) describing the DNA binding motif of each DBD, when available.**PMW IDs/motifs were assigned to each DBD (denoted as DBD1 and DBD2) as indicated in Methods.

**Supplementary File 5. Manually curated TFome of *B. cinerea* and *T. atroviride* with and without Position Weight Matrix (PWM) describing their respective DNA binding motifs.**PMW IDs/motifs were assigned to *B. cinerea* and *T. atroviride*TFs based on the CisBP “Protein Scan” web tool or the OrthoFinder analysis, as indicated in Methods. For each organism, TF gene ID, motif/PMW ID, motif/PMW TF Family Score, and the motif/PMW species are indicated.

**Supplementary File 6. *B. cinerea* regulatory interactions predicted by GENIE3.**

**Supplementary File 7. *T. atroviride*** **regulatory interactions predicted by GENIE3.**

**Supplementary File 8. Global gene expression dataset used for the GENIE3 GRN inference algorithm.**Each SRR file and respective metadata is provided for *B. cinerea*and *T. atroviride*. The reported information was gathered from the Sequence Read Archive (SRA) NCBI database.

**Supplementary File 9. *B. cinerea* gene regulatory networks.**The FIMO-only GRN, as well as the reference (FIMO+GENIE3) GRN, are provided.

**Supplementary File 10. *B. cinerea* context-specific gene regulatory networks.**The light GRN, as well as the confrontation-specific GRN, are provided.

**Supplementary File 11. *T. atroviride* gene regulatory networks.**The FIMO-only GRN, as well as the reference (FIMO+GENIE3) GRN, are provided.

**Supplementary File 12. *T. atroviride* context-specific gene regulatory networks.**The light GRN, as well as the confrontation-specific GRN, are provided.

**Supplementary File 13. Differentially expressed genes were determined under constant illumination for *B. cinerea*and *T. atroviride.***

**Supplementary File 14. Differentially expressed genes determined in the *Botrytis*-*Trichoderma* confrontation assay.**
